# Comprehensive assessment of the association between *XPC* rs2228000 and cancer susceptibility based on 26835 cancer cases and 37069 controls

**DOI:** 10.1042/BSR20192452

**Published:** 2019-12-04

**Authors:** Yingqi Dai, Zhonghua Song, Jinqing Zhang, Wei Gao

**Affiliations:** 1 Department of Breast Surgery, Shandong Provincial Third Hospital, Shandong 250031, P.R. China; 2 Department of Oncology, Shandong Provincial Third Hospital, Shandong 250031, P.R. China; 3 Department of Interventional Therapy, Tianjin Medical University Cancer Institute and Hospital, National Clinical Research Center for Cancer, Tianjin 300060, P.R. China; 4 Key Laboratory of Cancer Prevention and Therapy, Tianjin 300060, P.R. China; 5 Tianjin’s Clinical Research Center for Cancer, Huan Hu West Road, Tianjin 300060, P.R. China

**Keywords:** cancer, rs2228000, susceptibility, XPC

## Abstract

**Objectives** In the present study, we examined available articles from online databases to comprehensively investigate the effect of the *XPC* (xeroderma pigmentosum complementation group C) rs2228000 polymorphism on the risk of different types of clinical cancer.

**Methods** We conducted a group of overall and subgroup pooling analyses after retrieving the data from four databases (updated till September 2019). The *P*-value of association, OR (odds ratios), and 95% CI (confidence interval) were calculated.

**Results** We selected a total of 71 eligible studies with 26835 cancer cases and 37069 controls from the 1186 retrieved articles. There is an enhanced susceptibility for bladder cancer cases under T vs. C [*P*=0.004; OR (95% CI) = 1.25 (1.07, 1.45)], TT vs. CC [*P*=0.001; 1.68 (1.25, 2.26)], CT+TT vs. CC [*P*=0.016; 1.26 (1.04, 1.53)], and TT vs. CC+ CT [*P*=0.001; 1.49 (1.18, 1.90)] compared with negative controls. Additionally, there is an increased risk of breast cancer under T vs. C, TT vs. CC and TT vs. CC+ CT (*P*<0.05, OR > 1). Nevertheless, there is a decreased risk of gastric cancer cases in China under T vs. C [*P*=0.020; 0.92 (0.85, 0.99)], CT vs. CC [*P*=0.001, 0.83 (0.73, 0.93)], and CT+TT vs. CC [*P*=0.003, 0.84 (0.76, 0.94)].

**Conclusions** The TT genotype of *XPC* rs2228000 may be linked to an increased risk of bladder and breast cancer, whereas the CT genotype is likely to be associated with reduced susceptibility to gastric cancer in the Chinese population.

## Introduction

The human *XPC* (xeroderma pigmentosum complementation group C) gene is located on chromosome 3p25 and contains 16 exons and 15 introns [1,2]. The human XPC protein with 940 amino acids, encoded by *XPC*, serves as an essential member within the NER (nucleotide excision repair) pathway [3–5]. The XPC protein is important for the early damage site recognition and DNA repair initiation of NER [3,6,7]. The abnormal expression of the XPC protein was also reportedly linked to the progression of the cancer [3,8].

Within the *XPC* gene, three common variants, including rs2228000 (C21151T) of exon 8, rs2228001 (A33512C) of exon 15, and poly-AT insertion/deletion polymorphism (PAT^−/+^) of intron 9, were identified [4,9–11]. *XPC* rs2228000 results in a substitution of alanine for valine in position 499 (Ala499Val), while rs2228001 leads to a transversion from lysine to glutamine in position 939 (Lys939Gln) [4,9–11]. The present study investigated the potential genetic role of nonsynonymous *XPC* rs2228000 in the risk of different clinical types of cancer by pooling published studies with inconclusive conclusions.

After retrieving these studies, only three previous meta-analyses with no more than 15 studies in 2008 [12–14] and one meta-analysis with 33 studies in 2013 [15] were performed to assess the genetic association of *XPC* rs2228000 and the risk of overall cancer. Thus, we enrolled more sample sizes (71 case–control studies) and utilized different analysis strategies for an updated comprehensive evaluation in 2019 through meta-analysis and TSA (trial sequential analysis).

## Materials and methods

### Case–control study identification

PRISMA (Preferred Reporting Items for Systematic Reviews and Meta-Analyses) was utilized for our pooling analysis. In September 2019, we used a series of search terms (shown in Supplementary Table S1) to retrieve from four databases [PubMed, Embase, CChia National Knowledge Infrastructure (CNKI)) and WOS (Web of Science)] to obtain potentially relevant articles. We also designed a group of criteria for the inclusion/exclusion and eligibility assessment of the article. Inclusion criteria were the following: (1) case/control studies; (2) cancer; (3) *XPC* rs2228000; and (4) genotypic frequency data within both the case and control groups. Exclusion criteria were the following: (1) review; (2) meeting abstract; (3) case reports or family data; (4) meta-analysis; (5) cell, mice, horse, or other species; (6) other gene, disease or variant; (7) lack of specific data; (8) lack of normal group; (9) not in line with HWE (Hardy–Weinberg equilibrium); and (10) cohort.

### Basic information collection

We extracted some basic information, including author name, publication year, country, race, genotypic frequency, cancer type, control source, genotyping assay, and sample size, from the selected eligible case–control studies. The *P*-value of HWE based on the genotypic distribution in the control group was calculated.

### Article quality assessment

We utilized two approaches, including the NOS (Newcastle–Ottawa quality assessment scale) system (Supplementary Table S2) [16,17] and the risk-of-bias score system (Supplementary Table S3) [18,19] for the assessment of article quality. The article with an NOS score > 5 and a risk-of-bias score > 9 was considered to be high quality.

### Pooling analysis

We used STATA software (Stata Corporation, U.S.A.) to perform the association test in the overall and subgroup meta-analysis, heterogeneity assessment, Begg’s/Egger’s tests (for the publication bias evaluation) and sensitivity analysis (for data stability assessment) [16,17]. The OR (odds ratio), 95% CI (confidence interval) and *P*-value in a series of association tests under the five genetic models, including T vs. C (allele), TT vs. CC (homozygote), CT vs. CC (heterozygote), CT+TT vs. CC (dominant), and TT vs. CC+CT (recessive), were obtained. In addition, six factors, including race, country, control source, article quality, genotyping assay, and cancer type, were considered in our subgroup analysis.

The high heterogeneity was considered when the *I^2^* value in the *I^2^* test was larger than 50% and the *P*-value in the Q statistical test was less than 0.05, which led to the use of the DerSimonian–Laird method of the random-effect model. If not, a Mantel–Haenszel method of a fixed-effect model was used for the relatively low heterogeneity between studies.

### False-positive report probability

Targeting the positive findings, we also calculated the false-positive report probability (FPRP) and statistical power, as suggested by Wacholder et al. [20]. During analysis, an FPRP cut-off value of 0.2, a power OR of 1.5, and different prior probability levels (0.25, 0.1, 0.01, 0.001, 0.0001) were established. After assessing the research status regarding the association between *XPC* rs2228000 and cancer risk and referencing the similar publications [21,22], the FPRP value of the positive results less than 0.2 under the prior probability level of 0.1 indicates a noteworthy outcome.

### TSA

We also performed the TSA test to evaluate whether further research was needed, referring to some similar publications [23–26]. For the TSA parameter, a type I error probability of 5%, a statistical test power of 80%, and a low bias-based risk ratio reduction were established. Trial Sequential Analysis Viewer software (http://www.ctu.dk/tsa/) was utilized.

## Results

### Identification of eligible studies

**Figure 1:**
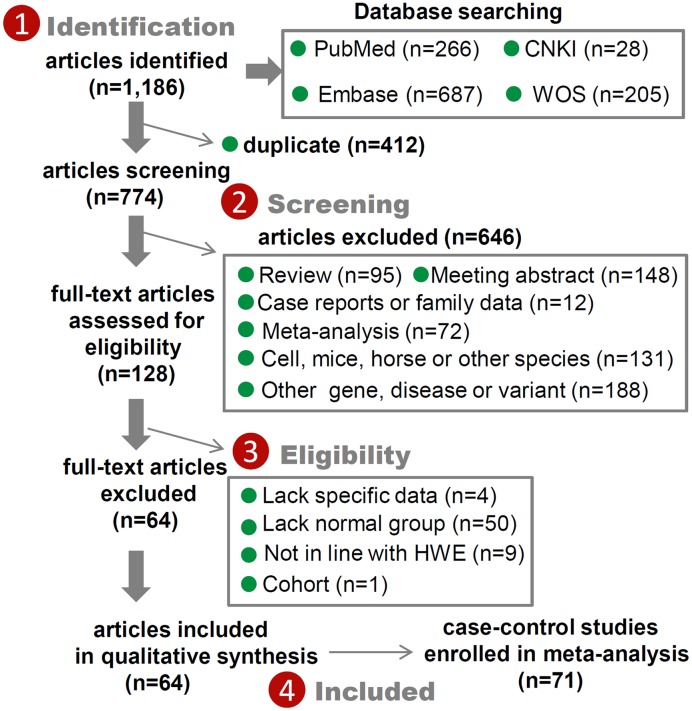
Selection process of eligible case–control studies

In total, we obtained 1186 potential eligible articles [PubMed (*n*=266), Embase [*n*=687], CNKI (*n*=28), and WOS (*n*=205)] and then ruled out another 412 duplicates and 646 improper articles according to our exclusion criteria (detailed information listed in Figure 1). Furthermore, we excluded 64 articles due to the question of ‘lack of specific data or normal group’, ‘not in line with HWE’ or ‘cohort’. Finally, we identified a total of 71 eligible case–control studies from the 64 retrieved articles [1,2,4,10,11,27–85] for pooling analysis. We summarized some basic information in Table 1 and presented the flow chart in Figure 1. All the genotypic distribution of the control group in all studies followed the principle of HWE. Although the NOS scores in all studies were larger than 5 (Supplementary Table S2), the risk-of-bias scores of nine articles (Supplementary Table S3) were less than 9.

**Table 1:** Basic information of the studies included in the meta-analysis

First author	Year	Country	Race	Cases			Cancer type	Control			Control source	Genotyping assay
				CC	CT	TT		CC	CT	TT		
**Al-Qadoori**	**2019**	Iraq	Asian	37	23	2	Bladder cancer	31	7	0	PB	Gene sequencing
**An**	**2007**	U.S.A.	Caucasian	445	293	91	HNSCC	454	342	58	HB	PCR-RFLP
**Bai**	**2007**	China	Asian	184	193	48	LAC	446	456	88	HB	TaqMan
		China	Asian	149	149	34	LSCC	446	456	88	HB	TaqMan
		China	Asian	31	25	8	SCLC	446	456	88	HB	TaqMan
**Broberg**	**2005**	Sweden	Caucasian	35	20	6	Bladder cancer	92	55	8	PB	MassARRAY
**Chen**	**2013**	China	Asian	45	60	26	Cervical cancer	101	118	38	HB	PCR-RFLP
**de Verdier**	**2010**	Sweden	Caucasian	138	138	35	Bladder cancer	196	124	10	PB	PCR-RFLP
**Doherty**	**2011**	U.S.A.	Mixed	411	257	49	Endometrial cancer	384	278	61	PB	PCR-RFLP/SNaPshot
**Dong**	**2008**	China	Asian	141	90	22	GCA	272	282	58	PB	PCR-RFLP
**Farnebo**	**2015**	Sweden	Caucasian	89	63	17	HNSCC	219	105	20	PB	PCR-RFLP
**Figl**	**2010**	Spain/Germany	Caucasian	626	477	81	Melanoma	670	516	88	PB	TaqMan
**Garcia**	**2006**	Spain	Caucasian	583	440	85	Bladder cancer	599	435	75	HB	SNP500Cancer
**Guo**	**2008**	China	Asian	156	133	38	ESCC	272	282	58	PB	PCR-RFLP
**He**	**2016**	China	Asian	201	198	51	Breast cancer	228	174	28	PB	MassARRAY
**He**	**2012**	China	Asian	104	90	16	Pancreatic cancer	106	85	22	PB	SNaPshot
**Hu**	**2005**	China	Asian	124	171	25	Lung cancer	158	145	19	PB	PCR-PIRA
**Hua**	**2016a**	China	Asian	432	531	178	CRC	429	583	161	PB	TaqMan
**Hua**	**2016b**	China	Asian	457	524	161	Gastric cancer	429	583	161	PB	TaqMan
**Huang**	**2006**	U.S.A.	Mixed	397	261	31	CRC	403	259	41	HB	SNP500Cancer
**Ibarrola**	**2011**	Spain	Caucasian	323	227	49	Melanoma	198	158	23	PB/HB	MassARRAY
**Jiao**	**2011**	China	Asian	127	177	30	GBC	163	146	20	HB	PCR-RFLP
**Jorgensen**	**2007**	U.S.A.	Caucasian	153	87	13	Breast cancer	157	104	14	PB	TaqMan
**Kim**	**2002**	Korea	Asian	104	102	12	Lung cancer	77	62	10	PB	PCR-RFLP
**Lee**	**2005**	Korea	Asian	113	84	13	LSCC	223	179	29	PB	PCR-RFLP
		Korea	Asian	79	58	4	LAC	223	179	29	PB	PCR-RFLP
		Korea	Asian	39	28	6	SCLC	223	179	29	PB	PCR-RFLP
**Li**	**2006**	U.S.A.	Caucasian	338	214	50	Melanoma	318	248	37	HB	PCR-RFLP
**Li**	**2014**	China	Asian	92	91	19	Gastric cancer	144	153	30	PB	PCR-RFLP
**Li**	**2010**	China	Asian	163	248	89	HCC	169	250	88	HB	TaqMan
**Liang**	**2018**	China	Asian	98	89	18	Pancreatic cancer	116	90	24	HB	SNaPshot
**Liu**	**2016**	China	Asian	444	351	96	Gastric cancer	424	408	95	HB	MassARRAY
**Liu**	**2012**	China	Asian	242	294	64	Bladder cancer	272	285	52	PB	PCR-RFLP
**Liu**	**2019**	China	Asian	178	159	54	Uterine leiomyoma	183	232	78	PB	Sequence Detection System
**Long**	**2010**	China	Asian	170	156	35	GAA	280	274	62	HB	TaqMan
**McWilliams**	**2008**	U.S.A.	Mixed	246	182	29	Pancreatic cancer	339	211	32	HB	SNPstream or Pyrosequencing
**Monroy**	**2011**	U.S.A.	Mixed	92	90	8	HL	137	71	10	PB	MassARRAY
**Na**	**2012**	China	Asian	213	124	23	Breast cancer	228	118	14	HB	MassARRAY
**Nigam**	**2019**	China	Asian	22	22	26	Oral cancer	69	145	83	PB	PCR-RFLP
**Ozgoz**	**2019**	Turkey	Caucasian	57	38	7	Breast cancer	67	26	7	PB	MassARRAY
**Pan**	**2009**	U.S.A	Caucasian	228	129	26	Esophageal cancer	251	178	21	PB	TaqMan
**Paszkowska**	**2015**	Poland	Caucasian	443	269	41	CRC	548	563	177	PB	MassARRAY/Taqman
**Paszkowska**	**2013**	Poland	Caucasian	245	240	34	Melanoma	548	563	177	PB	MassARRAY
**Perez**	**2013**	U.S.A.	Caucasian	0	63	115	Breast cancer	21	131	203	PB	TaqMan
**Ravegnini**	**2016**	Italy	Caucasian	42	34	5	GIST	90	45	12	PB	TaqMan
**Roberts**	**2011**	U.S.A.	Mixed	167	100	18	Breast cancer^1^	317	193	40	PB	MassARRAY
		U.S.A.	Mixed	437	273	48	Breast cancer^2^	793	478	72	PB	MassARRAY
**Sak**	**2006**	U.K.	Mixed	279	202	57	Bladder cancer	317	210	38	PB/HB	TaqMan
**Sakoda**	**2012**	U.S.A.	Caucasian	401	299	43	Lung cancer	822	566	87	PB	GoldenGate/Taqman
**Sankhwar**	**2016**	India	Asian	52	113	69	Bladder cancer	87	112	59	PB	PCR-RFLP/gene sequencing
**Santos**	**2013**	Portugal	Caucasian	47	55	4	Thyroid cancer	95	98	19	HB	PCR-RFLP
**Shen**	**2006**	U.S.A.	Caucasian	96	50	9	Breast cancer	91	55	5	PB	TaqMan
**Shen**	**2008**	U.S.A.	Mixed	614	385	62	Breast cancer	632	417	56	PB	Fluorescence polarization
**Shen**	**2005**	China	Asian	56	47	13	Lung cancer	50	47	13	PB	TaqMan
**Slyskova**	**2012**	Czech Republic	Caucasian	36	24	9	CRC	37	24	3	PB	PCR-RFLP
**Smith**	**2008**	U.S.A.	Caucasian	178	116	23	Breast cancer	211	161	29	PB	MassARRAY
		U.S.A.	Others	44	7	1	Breast cancer	61	14	0	PB	MassARRAY
**Steck**	**2014**	U.S.A.	Others	175	51	2	CRC	276	47	0	PB	MassARRAY
		U.S.A.	Caucasian	177	104	22	CRC	293	207	35	PB	MassARRAY
**Tang**	**2011**	China	Asian	40	55	14	ALL	80	74	15	PB	MassARRAY
**Weiss**	**2005**	U.S.A.	Mixed	211	129	31	Endometrial cancer	213	166	41	PB	SNaPshot
**Wu**	**2011a**	China	Asian	172	195	52	CRC	315	406	117	PB	PCR-RFLP
**Wu**	**2011b**	China	Asian	65	86	22	Breast cancer	69	85	16	PB	PCR-RFLP
**Yang**	**2012**	China	Asian	197	322	99	Breast cancer	235	312	75	PB	PCR-RFLP
**Yang**	**2008**	China	Asian	52	73	28	NPC	76	79	13	PB	PCR-RFLP
**Zhao**	**2018**	China	Asian	46	35	8	Ovarian cancer	127	175	54	PB	TaqMan
**Zheng**	**2016**	China	Asian	111	108	34	Neuroblastoma	205	250	76	PB	TaqMan
**Zhou**	**2008**	China	Asian	103	78	27	Ovarian cancer	118	95	18	PB	PCR-RFLP
**Zhu**	**2018**	China	Asian	64	59	22	Nneuroblastoma	205	250	76	PB	TaqMan
**Zhu**	**2008**	China	Asian	110	60	18	ESCC	83	88	32	PB	PCR-RFLP
**Zhu**	**2007**	U.S.A.	Caucasian	323	193	30	Bladder cancer	310	215	24	HB	TaqMan

Abbreviations: ALL, acute lymphoblastic leukemia; CRC, colorectal cancer; ESCC, esophageal squamous cell carcinoma; GAA, gastric antrum adenocarcinoma; GBC, primary gallbladder adenocarcinoma; GCA, gastric cardiac adenocarcinoma; GIST, gastrointestinal stromal tumour; HB, hospital-based; HCC, hepatocellular carcinoma; HL, Hodgkin lymphoma; HNSCC, head and neck squamous cell carcinoma; LAC, lung adenocarcinoma; LSCC, lung squamous cell carcinoma; NPC, nasopharyngeal cancer; PB, population-based; PCR, polymerase chain reaction; PIRA, primer-introduced restriction analysis; RFLP, restriction fragment length polymorphism; SCLC, Small cell lung carcinoma; SNP, single nucleotide polymorphism. ^1^ Premenopausal. ^2^ Postmenopausal.

### Overall meta-analysis

As shown in Table 2, our overall meta-analysis included a total of 71 studies with 26835 cases and 37069 controls. We observed high between-study heterogeneity (Table 2, all *I^2^* > 50%, *P*_heterogeneity_<0.001) and thus utilized the random-effect model for the pooling analysis. After pooling the different studies together, we only detected an increased risk of overall cancers under the TT vs. CC+CT model [Table 2, *P_association_*=0.023, OR = 1.11, 95% CI = (1.01, 1.22)] but not other models (all *P_association_*>0.05). These results indicated that *XPC* rs2228000 does not seem to be statistically associated with susceptibility to cancer.

**Table 2:** Meta-analysis of *XPC* rs2228000 and overall cancer risk

Genetic model	Sample size		Association		Heterogeneity		Publication bias	
	Study	Case/control	*P_association_*	OR (95% CI)	*I^2^*	*P_heterogeneity_*	*P_Begg_*	*P_Egger_*
**T vs. C**	71	26835/37069	0.218	1.03 (0.98,1.09)	72.2%	<0.001	0.079	0.031
**TT vs. CC**	71	26835/37069	0.090	1.10 (0.99,1.23)	64.6%	<0.001	0.124	0.065
**CT vs. CC**	71	26835/37069	0.588	0.98 (0.93,1.04)	59.1%	<0.001	0.093	0.046
**CT+TT vs. CC**	71	26835/37069	0.793	1.01 (0.95,1.07)	68.0%	<0.001	0.069	0.023
**TT vs. CC+ CT**	71	26835/37069	0.023	1.11 (1.01,1.22)	54.1%	<0.001	0.493	0.230

Abbreviations: *P_association_*, *P*-value in the association test; *P_heterogeneity_*, *P*-value in the heterogeneity test; *P_Begg_*, *P*-value in Begg’s test; *P_Egger_*, *P*-value in Egger’s test.

### Subgroup analysis

**Figure 2:**
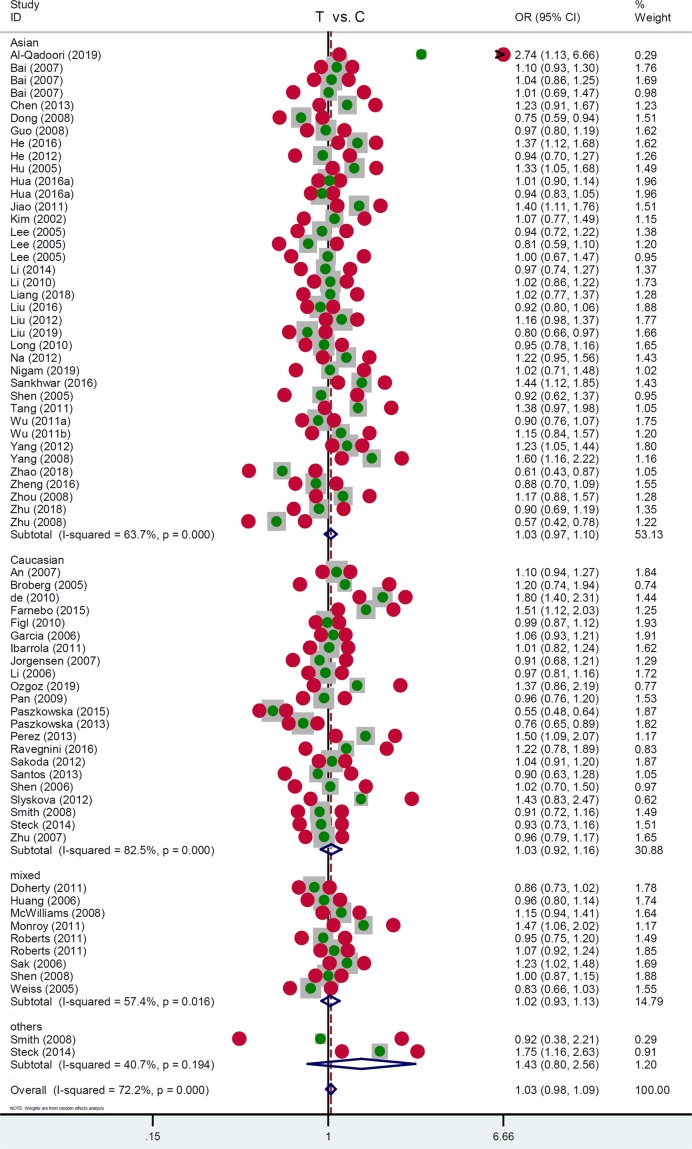
Subgroup analysis data by the factor of race under the T vs. C model

Next, we performed a series of subgroup analyses by the factors of race, control source, country, article quality, and genotyping assay. As shown in Table 3, a total of 38 studies (12118 cases/18124 controls) were included for the subgroup analysis of ‘Asian’, while 22 studies with 9371 cases and 12338 controls were included for the ‘Caucasian’ subgroup. We did not observe a significant difference between cancer cases and negative controls under the most genetic models (Table 3, *P_association_*>0.05), only apart from the Asian subgroup under the TT vs. CC+CT model [*P_association_*=0.005, OR = 1.13, 95% CI = (1.04, 1.23)]. Within the subgroup analysis by the factor or control source (PB/HB (population/hospital-based)), an increased risk of cancer was only detected in the ‘HB’ subgroup under TT vs. CC [Table 3, *P_association_*=0.010, OR = 1.17, 95% CI = (1.04,1.32)] and TT vs. CC+CT [*P_association_*=0.006, OR = 1.18, 95% CI = (1.05, 1.32)] models but not others (*P_association_*>0.05). Similarly, we observed negative results in the majority of the subgroup analyses by country, article quality and genotyping assay (Supplementary Table S4). As examples, we presented the forest plots of the subgroup analysis data by the factor of race (Figure 2), control source (Supplementary Figure S1), country (Supplementary Figure S2), article quality (Supplementary Figure S3), and genotyping assay (Supplementary Figure S4) under the T vs. C model.

**Table 3:** Subgroup analysis data by the factors of race and control source

Genetic model	Subgroup	Sample size		Association	
		Study	Case/Control	*P_association_*	OR (95% CI)
**T vs. C**	Asian	38	12118/18124	0.360	1.03 (0.97, 1.10)
	Caucasian	22	9371/12338	0.572	1.03 (0.92, 1.16)
	PB	52	17758/25317	0.447	1.03 (0.96, 1.10)
	HB	17	7940/10808	0.109	1.04 (0.99, 1.09)
**TT vs. CC**	Asian	38	12118/18124	0.091	1.11 (0.98, 1.25)
	Caucasian	22	9371/12338	0.329	1.15 (0.87, 1.53)
	PB	52	17758/25317	0.397	1.07 (0.92, 1.23)
	HB	17	7940/10808	0.010	1.17 (1.04, 1.32)
**CT vs. CC**	Asian	38	12118/18124	0.554	0.98 (0.90, 1.06)
	Caucasian	22	9371/12338	0.483	0.96 (0.86, 1.07)
	PB	52	17758/25317	0.612	0.98 (0.91, 1.06)
	HB	17	7940/10808	0.857	0.99 (0.92, 1.07)
**CT+TT vs. CC**	Asian	38	12118/18124	0.948	1.00 (0.92, 1.09)
	Caucasian	22	9371/12338	0.905	0.99 (0.88, 1.12)
	PB	52	17758/25317	0.965	1.00 (0.92, 1.09)
	HB	17	7940/10808	0.581	1.02 (0.95, 1.09)
**TT vs. CC+ CT**	Asian	38	12118/18124	0.005	1.13 (1.04, 1.23)
	Caucasian	22	9371/12338	0.293	1.14 (0.89, 1.45)
	PB	52	17758/25317	0.219	1.08 (0.96, 1.21)
	HB	17	7940/10808	0.006	1.18 (1.05, 1.32)

Abbreviations: PB, population-based; *P_association_, P*-value in the association test.

**Figure 3:**
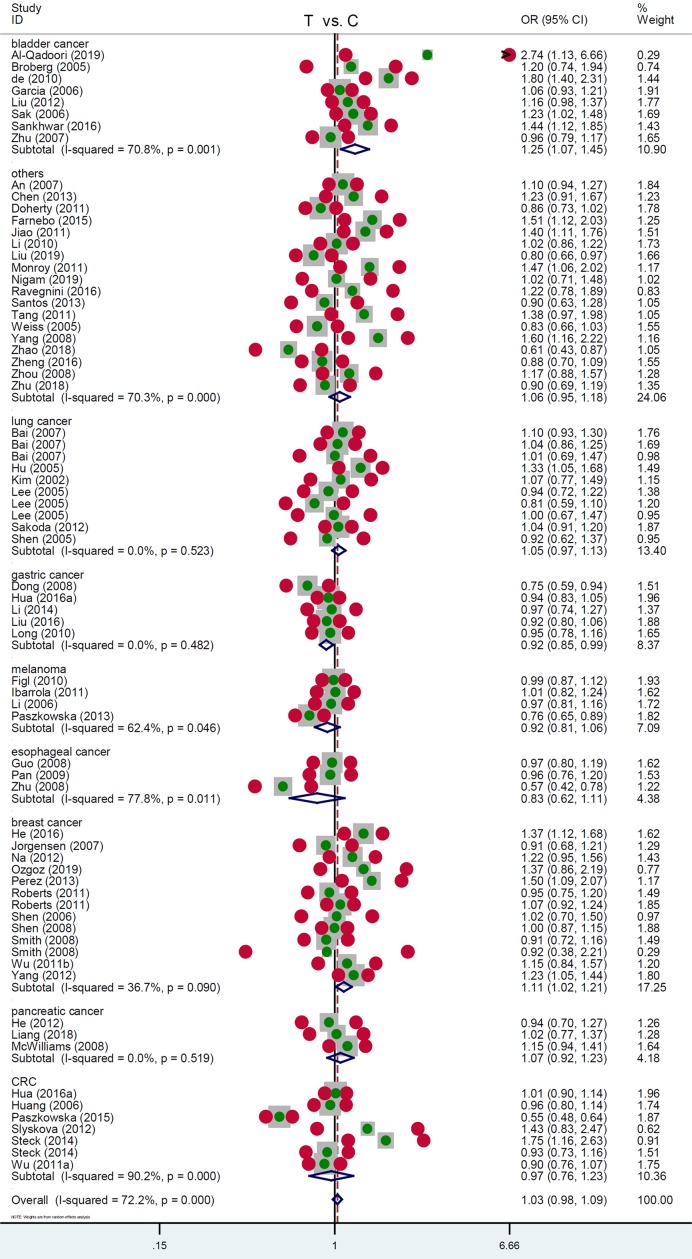
Subgroup analysis data by the factor of cancer type under the T vs. C model

Additionally, we performed a subgroup analysis using the specific cancer type. As shown in Table 4, in the subgroup of ‘bladder cancer’ with 3460 cases and 3613 controls, enhanced susceptibility was detected in bladder cancer cases under T vs. C [Table 4, *P_association_*=0.004, OR = 1.25, 95% CI = (1.07, 1.45)], TT vs. CC [*P_association_*=0.001, OR = 1.68, 95% CI = (1.25, 2.26)], CT+TT vs. CC [*P_association_*=0.016, OR = 1.26, 95% CI = (1.04, 1.53)], TT vs. CC+ CT [*P_association_*= 0.001, OR = 1.49, 95% CI = (1.18, 1.90)] compared with the negative controls. Additionally, there is an increased risk of breast cancer under T vs. C [Table 4, *P_association_*=0.018, OR = 1.11, 95% CI = (1.02, 1.21)], TT vs. CC [*P_association_*=0.003, OR = 1.33, 95% CI = (1.10, 1.60)], and TT vs. CC+ CT [*P_association_*= 0.001, OR = 1.29, 95% CI = (1.12, 1.48)]. Nevertheless, we observed a decreased risk of gastric cancer in the Chinese population under T vs. C [Table 4, *P_association_*=0.020, OR = 0.92, 95% CI = (0.85, 0.99)], CT vs. CC [*P_association_*=0.001, OR = 0.83, 95% CI = (0.73, 0.93)], CT+TT vs. CC [*P_association_*=0.003, OR = 0.84, 95% CI = (0.76, 0.94)]. The relevant forest plots under different genetic models are presented in Figure 3 (T vs. C), Supplementary Figure S5 (TT vs. CC), Supplementary Figure S6 (CT vs. CC), Supplementary Figure S7 (CT+TT vs. CC), and Supplementary Figure S8 (TT vs. CC+ CT).

**Table 4:** Subgroup analysis data by the factors of specific cancer type

Genetic model	Subgroup	Sample size		Association	
		Study	Case/Control	*P_association_*	OR (95% CI)
**T vs. C**	Bladder cancer	8	3460/3613	0.004	1.25 (1.07, 1.45)
	Lung cancer	10	2642/6319	0.222	1.05 (0.97, 1.13)
	Gastric cancer	5	2849/3655	0.020	0.92 (0.85, 0.99)
	Melanoma	4	2904/3544	0.250	0.92 (0.81, 1.06)
	Esophageal cancer	3	898/1265	0.210	0.83 (0.62, 1.11)
	Breast cancer	13	4762/5937	0.018	1.11 (1.02, 1.21)
	Pancreatic cancer	3	872/1025	0.380	1.07 (0.92, 1.23)
	CRC	7	3602/4924	0.776	0.97 (0.76, 1.23)
**TT vs. CC**	Bladder cancer	8	3460/3613	0.001	1.68 (1.25, 2.26)
	Lung cancer	10	2642/6319	0.252	1.11 (0.93, 1.34)
	Gastric cancer	5	2849/3655	0.361	0.93 (0.78, 1.09)
	Melanoma	4	2904/3544	0.697	0.90 (0.55, 1.50)
	Esophageal cancer	3	898/1265	0.724	0.89 (0.46, 1.71)
	Breast cancer	13	4762/5937	0.003	1.33 (1.10, 1.60)
	Pancreatic cancer	3	872/1025	0.952	0.99 (0.69, 1.41)
	CRC	7	3602/4924	0.588	0.87 (0.52, 1.45)
**CT vs. CC**	Bladder cancer	8	3460/3613	0.069	1.17 (0.99, 1.39)
	Lung cancer	10	2642/6319	0.368	1.05 (0.95, 1.16)
	Gastric cancer	5	2849/3655	0.001	0.83 (0.73, 0.93)
	Melanoma	4	2904/3544	0.157	0.93 (0.83, 1.03)
	Esophageal cancer	3	898/1265	0.013	0.73 (0.57, 0.94)
	Breast cancer	13	4762/5937	0.418	1.04 (0.94, 1.16)
	Pancreatic cancer	3	872/1025	0.128	1.16 (0.96, 1.40)
	CRC	7	3602/4924	0.405	0.91 (0.73, 1.13)
**CT+TT vs. CC**	Bladder cancer	8	3460/3613	0.016	1.26 (1.04, 1.53)
	Lung cancer	10	2642/6319	0.282	1.05 (0.96, 1.16)
	Gastric cancer	5	2849/3655	0.003	0.84 (0.76, 0.94)
	Melanoma	4	2904/3544	0.088	0.92 (0.83, 1.01)
	Esophageal cancer	3	898/1265	0.065	0.74 (0.54, 1.02)
	Breast cancer	13	4762/5937	0.175	1.08 (0.97, 1.21)
	Pancreatic cancer	3	872/1025	0.182	1.13 (0.94, 1.36)
	CRC	7	3602/4924	0.563	0.93 (0.71, 1.20)
**TT vs. CC+ CT**	Bladder cancer	8	3460/3613	0.001	1.49 (1.18, 1.90)
	Lung cancer	10	2642/6319	0.293	1.10 (0.92, 1.32)
	Gastric cancer	5	2849/3655	0.834	1.02 (0.87, 1.19)
	Melanoma	4	2904/3544	0.826	0.94 (0.56, 1.59)
	Esophageal cancer	3	898/1265	0.889	1.04 (0.61, 1.78)
	Breast cancer	13	4762/5937	0.001	1.29 (1.12, 1.48)
	Pancreatic cancer	3	872/1025	0.669	0.94 (0.66, 1.31)
	CRC	7	3602/4924	0.682	0.91 (0.58, 1.43)

Abbreviations: CRC, colorectal cancer; *P_association_, P*-value in the association test.

Moreover, we performed subgroup analysis data for different system cancers. As shown in Supplementary Table S5 and Figure S9 (forest plot data under the allelic model), we observed the same result in the subgroup of ‘urinary system cancer’ as the subgroup of ‘bladder cancer’. There is a reduced cancer risk in the subgroup of ‘reproductive system cancer’ under the models of CT vs. CC [*P_association_*=0.006, OR = 0.81, 95% CI = (0.70, 0.94)] and CT+TT vs. CC [*P_association_*=0.041, OR = 0.82, 95% CI = (0.68, 0.99)] and an increased risk in the subgroup of ‘head and neck cancer’ under the TT vs. CC+CT [*P_association_*=0.024, OR = 1.58, 95% CI = (1.06, 2.34)]. However, no positive association was observed in other subgroups (Supplementary Table S5, *P_association_*>0.05).

The above results indicated that the TT genotype of *XPC* rs2228000 seems to be related to a high risk of bladder and breast cancer, whereas the CT genotype is more likely to be associated with reduced susceptibility to gastric cancer in the Chinese population.

### Publication bias/sensitivity

**Figure 4:**
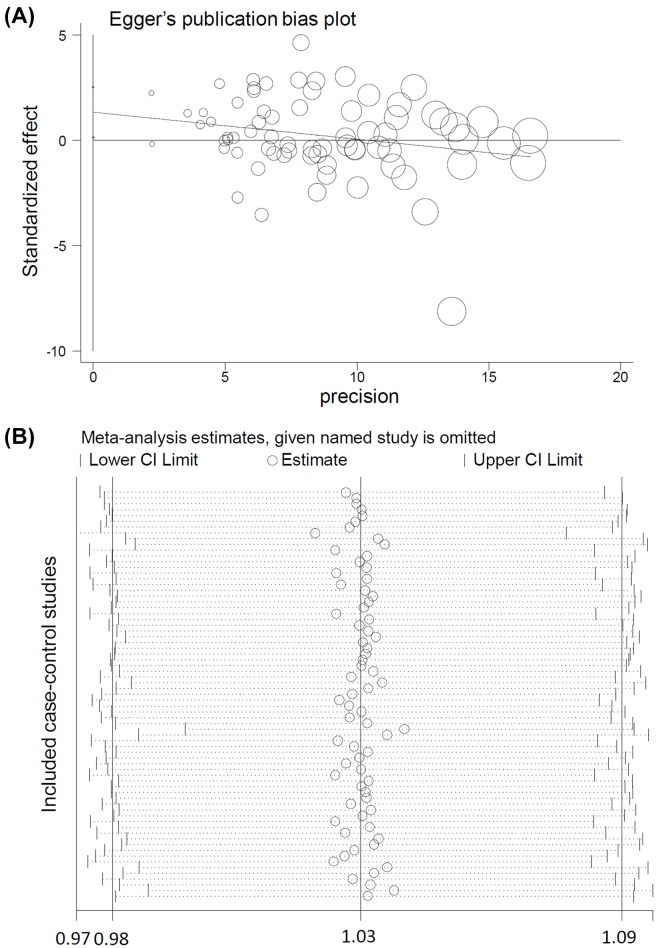
Egger’s test plot and the sensitivity analysis data under the T vs. C model (**A**) Egger’s test; (**B**) sensitivity analysis data.

As shown in Table 2, we did not observe a notable publication bias among these comparisons, in that all the *P_Begg_*>0.05, *P_Egger_*>0.05 apart from the *P_Egger_*=0.031 (T vs. C), *P_Egger_*=0.046 (CT vs. CC), *P_Egger_*=0.023 (CT+TT vs. CC). Figure 4A presents the publication bias plot of Egger’s test under the T vs. C model. In addition, as shown in Figure 4B (allelic model data as example), we also observed relatively stable pooling data through the performance of sensitivity analyses.

### FPRP/TSA

An FPRP test was conducted to confirm the above positive findings for bladder, breast, and gastric cancers. The FPRP values of positive results at different prior probability levels are shown in Supplementary Table S6. We found that at a prior probability of 0.1 with an OR of 1.5, all the FPRP values were less than 0.2 (Supplementary Table S6, FPRP = 0.028, T vs. C; FPRP = 0.023, TT vs. CC; FPRP = 0.155, CT+TT vs. CC; FPRP = 0.022, TT vs. CC+ CT), indicating a noteworthy association between *XPC* rs2228000 and the risk of bladder cancer. Similar true positive associations were observed for breast and gastric cancer (Supplementary Table S6, all FPRP < 0.02) at a prior probability of 0.1.

**Figure 5:**
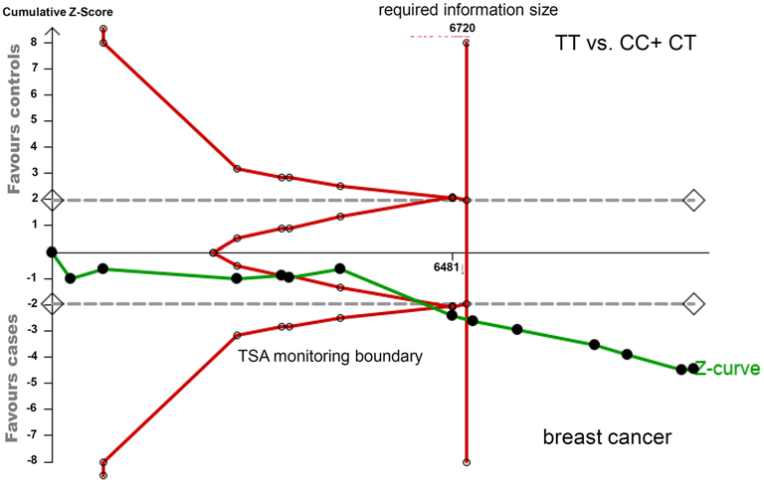
TSA for the association between *XPC* rs2228000 and the risk of breast cancer under the TT vs. CC+CT model

In addition, we also performed the TSA test to assess the robustness of our significant findings. As shown in the TSA data of breast cancer under the TT vs. CC+CT model (Figure 5) and gastric cancer under the CT+TT vs. CC models (Supplementary Figure S10), we found that the cumulative number of participants (Z-curve) met the TSA monitoring boundary and required information size. With regard to the bladder cancer under the TT vs. CC+CT model (Supplementary Figure S11), the cumulative Z-curve crossed with the TSA monitoring boundary, even though it did not reach the required information size. These data therefore indicated the robustness of our conclusions.

## Discussion

There is a controversial conclusion regarding the genetic impacts of the *XPC* rs2228000 SNP in the risk of clinical cancer diseases in different publications. For example, *XPC* rs2228000 was reportedly related to susceptibility to bladder cancer cases in Iraq [27], Sweden [30], or India [67] but not the U.S.A. [85] or Spain [35]. Likewise, *XPC* rs2228000 was also significantly associated with the risk of breast cancer in a Chinese population [37,78] but not Caucasians or African-Americans in the U.S.A. [72]. Although several meta-analyses of *XPC* rs2228000 and certain specific cancer types exist [86–92], differences in study enrolment, data extraction, analysis strategy, and result descriptions were observed. We thus conducted a meta-analysis and TSA for a comprehensive assessment regarding the genetic influence of the *XPC* rs2228000 in the risk of various types of cancer, including bladder cancer, lung cancer, gastric cancer, melanoma, esophageal cancer, breast cancer, pancreatic cancer, and colorectal cancer.

Only three prior meta-analyses with fewer than 15 studies in 2008 [12–14] and one meta-analysis with 33 articles in 2013 [15] were reported to detect the genetic association between *XPC* rs2228000 and overall cancer risk. In our study, we retrieved four databases (updated till September 2019) to include the potential publication for the pooling analysis. After employing our strict screen strategy, we finally included 64 eligible articles, which contained 71 case–control studies, for the overall meta-analysis and the following subgroup analyses by the factors of race, country, control source, article quality, genotyping assay, and cancer type. Five genetic models, including allelic, homozygotic, heterozygotic, dominant, and recessive models, were utilized. We excluded the improper studies according to the strict requirement of full genotype frequency data and the HWE principle. For instance, there are a total of 33 articles with 14877 cases and 17888 controls [1,28,30–32,36,39,42,44,45,47,48,50,54–56,60,64–66,68–70,72,75–79,82,93–95] for the prior meta-analysis of He et al. in 2013 [15]. In this study, we excluded two articles regarding bladder cancer [95] and cutaneous melanoma [94] because the genotype distribution in the control group is not in line with the HWE, and we added 32 other published articles [4,10,11,27,29,33–35,37,38,40,41,43,46,49,51,52,57–59,61–63,67,71,73,74,80,81,83–85]. Our pooling data from eight case–control studies showed the genetic correlation between *XPC* rs2228000 and increased risk of bladder cancer under the allelic, homozygotic, heterozygotic, dominant, and recessive models, which is partly consistent with the positive data of He et al. (2013) [15] under the homozygotic and recessive models from four case–control studies. A similar result was obtained for breast cancer, even though four new case–control studies were added, compared with the pooling results of He et al. (2013) [15]. Moreover, we provided assessment evidence regarding the potential impact of *XPC* rs2228000 on the reduced susceptibility to gastric cancer in the Chinese population. Nevertheless, we did not detect a significant association between *XPC* rs2228000 and other types of cancer, such as lung cancer, melanoma, pancreatic cancer, or colorectal cancer.

In our study, we performed the FPRP test with a prior probability of 0.1 and an FPRP threshold of 0.2 to check whether the positive findings of breast, bladder, and gastric cancers are noteworthy, considering the potential presence of ‘false positives’. After the FPRP estimation, the genetic association between *XPC* rs2228000 and the risk of bladder, breast, and gastric cancers risk remain significant at the prior probability level of 0.1. Furthermore, we observed the robustness of our conclusions through the performance of TSA test and sensitivity analyses and the absence of large publication bias by Begg’s/Egger’s test.

Despite these findings, some limitations to this research may still influence the statistical power of analyses of certain types of cancer. Although more than 70 case–control studies were enrolled in the overall meta-analysis, small sample sizes were still included in some subgroup analyses. For example, only two case–control studies [56,74] were included for the subgroup of ‘blood system cancer’, while only two studies [81,83] were enrolled for ‘nervous system cancer’. Therefore, we still cannot rule out the possible genetic role played by *XPC* rs2228000 in the risk of cancers of the blood or nervous systems. A similar uncertainty also exists in the subgroup analysis of ‘lung cancer’, ‘melanoma’, ‘esophageal cancer’, ‘pancreatic cancer’ and ‘CRC’.

We observed clear between-study heterogeneity, even if articles with low quality are removed. Regarding the available sample size, more factors, such as gender, age, environmental exposure, drinking/smoking status, tumor situations, characteristics, antiepileptic agents, or drug resistance, should be adjusted in future pooling analyses. It would be valuable to carry out an integrated analysis to evaluate the combined role of more *XPC* polymorphic loci (e.g., rs2228001, PAT^−/+^) in susceptibility to different types of cancer based on the available evidence.

## Conclusions

In general, the TT genotype of *XPC* rs2228000 may be linked to an increased risk of bladder and breast cancers, whereas the CT genotype is more likely to be associated with a reduced susceptibility to gastric cancer in the Chinese population. Considering the limitations of our study, we need to analyze more publications to verify the genetic impact of *XPC* rs2228000 in other types of cancer.

## Supplementary Material

Supplementary Figures S1-S11 and Tables S1-S6

## Abbreviations

CI: confidence interval
CNKI: China National Knowledge Infrastructure
FPRP: false-positive report probability
HB: hospital-based
HWE: Hardy–Weinberg equilibrium
NER: nucleotide excision repair
NOS: Newcastle–Ottawa quality assessment Scale
OR: odds ratio
PAT^−/+^: poly-AT insertion/deletion polymorphism
TSA: trial sequential analysis
WOS: Web of Science
XPC: xeroderma pigmentosum complementation group C
