# Supplementary material for: Comprehensive assessment of the association between XPC rs2228000 and cancer susceptibility based on 26835 cancer cases and 37069 controls
Source: Biosci Rep. 2019 Dec 4;39(12):BSR20192452. doi: 10.1042/BSR20192452 (PMC6893172; doi:10.1042/BSR20192452)
Supplement: Supplementary Figures S1-S11 and Tables S1-S6 [file BSR-2019-2452_supp.pdf]

Supplemental data

Figure S1. Subgroup analysis data by the factor of control source under the T vs. C model.

Figure S2. Subgroup analysis data by the factor of country under the T vs. C model.

Figure S3. Subgroup analysis data by the factor of article quality under the T vs. C model.

Figure S4. Subgroup analysis data by the factor of genotyping assay under the T vs. C model.
Figure S5. Subgroup analysis data by the factor of cancer type under the TT vs. CC model.

Figure S6. Subgroup analysis data by the factor of cancer type under the CT vs. CC model.

Figure S7. Subgroup analysis data by the factor of cancer type under the CT+TT vs. CC model.

Figure S8. Subgroup analysis data by the factor of cancer type under the TT vs. CC+ CT model.

Figure S9. Subgroup analysis data of different system cancers under the T vs. C model.

Figure S10. Trial sequential analysis for the association between *XPC* rs2228000 and the risk of gastric cancer under the CT+TT vs. CC model.

Figure S11. Trial sequential analysis for the association between *XPC* rs2228000 and the risk of bladder cancer under the TT vs. CC+CT model.

Table S1. Database search strategy in our study.

Table S2. Article quality assessment using the NOS system.

Table S3. Article quality assessment using the risk-of-bias score system.

Table S4. Subgroup analysis data by the factors of country, genotyping assay and article quality.

Table S5. Subgroup analysis data of different system cancers.

Table S6. False-positive report probability values for the association between *XPC* rs2228000 and the risk of bladder, gastric and breast cancer.

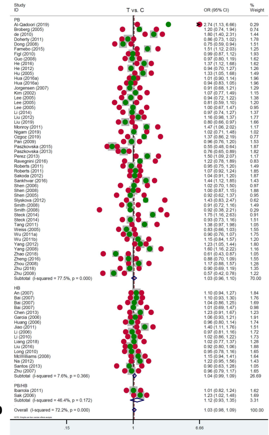

Study
ID
T vs. C
OR (95% CI)
%
Weight
PB
Al-Qadoori (2019) 2.74 (1.13, 6.66) 0.29
Broberg (2005) 1.20 (0.74, 1.94) 0.74
de (2010) 1.80 (1.40, 2.31) 1.44
Doherty (2011) 0.86 (0.73, 1.02) 1.78
Dong (2008) 0.75 (0.59, 0.94) 1.51
Farnebo (2015) 1.51 (1.12, 2.03) 1.25
Figl (2010) 0.99 (0.87, 1.12) 1.93
Guo (2008) 0.97 (0.80, 1.19) 1.62
He (2016) 1.37 (1.12, 1.68) 1.62
He (2012) 0.94 (0.70, 1.27) 1.26
Hu (2005) 1.33 (1.05, 1.68) 1.49
Hua (2016a) 1.01 (0.90, 1.14) 1.96
Hua (2016a) 0.94 (0.83, 1.05) 1.96
Jorgensen (2007) 0.91 (0.68, 1.21) 1.29
Kim (2002) 1.07 (0.77, 1.49) 1.15
Lee (2005) 0.94 (0.72, 1.22) 1.38
Lee (2005) 0.81 (0.59, 1.10) 1.20
Lee (2005) 1.00 (0.67, 1.47) 0.95
Li (2014) 0.97 (0.74, 1.27) 1.37
Liu (2012) 1.16 (0.98, 1.37) 1.77
Liu (2019) 0.80 (0.66, 0.97) 1.66
Monroy (2011) 1.47 (1.06, 2.02) 1.17
Nigam (2019) 1.02 (0.71, 1.48) 1.02
Ozgoz (2019) 1.37 (0.86, 2.19) 0.77
Pan (2009) 0.96 (0.76, 1.20) 1.53
Paszkowska (2015) 0.55 (0.48, 0.64) 1.87
Paszkowska (2013) 0.76 (0.65, 0.89) 1.82
Perez (2013) 1.50 (1.09, 2.07) 1.17
Ravegnini (2016) 1.22 (0.78, 1.89) 0.83
Roberts (2011) 0.95 (0.75, 1.20) 1.49
Roberts (2011) 1.07 (0.92, 1.24) 1.85
Sakoda (2012) 1.04 (0.91, 1.20) 1.87
Sankhwar (2016) 1.44 (1.12, 1.85) 1.43
Shen (2006) 1.02 (0.70, 1.50) 0.97
Shen (2008) 1.00 (0.87, 1.15) 1.88
Shen (2005) 0.92 (0.62, 1.37) 0.95
Slyskova (2012) 1.43 (0.83, 2.47) 0.62
Smith (2008) 0.91 (0.72, 1.16) 1.49
Smith (2008) 0.92 (0.38, 2.21) 0.29
Steck (2014) 1.75 (1.16, 2.63) 0.91
Steck (2014) 0.93 (0.73, 1.16) 1.51
Tang (2011) 1.38 (0.97, 1.98) 1.05
Weiss (2005) 0.83 (0.66, 1.03) 1.55
Wu (2011a) 0.90 (0.76, 1.07) 1.75
Wu (2011b) 1.15 (0.84, 1.57) 1.20
Yang (2012) 1.23 (1.05, 1.44) 1.80
Yang (2008) 1.60 (1.16, 2.22) 1.16
Zhao (2018) 0.61 (0.43, 0.87) 1.05
Zheng (2016) 0.88 (0.70, 1.09) 1.55
Zhou (2008) 1.17 (0.88, 1.57) 1.28
Zhu (2018) 0.90 (0.69, 1.19) 1.35
Zhu (2008) 0.57 (0.42, 0.78) 1.22
Subtotal (I-squared = 77.5%, p = 0.000) 1.03 (0.96, 1.10) 70.00
HB
An (2007) 1.10 (0.94, 1.27) 1.84
Bai (2007) 1.10 (0.93, 1.30) 1.76
Bai (2007) 1.04 (0.86, 1.25) 1.69
Bai (2007) 1.01 (0.69, 1.47) 0.98
Chen (2013) 1.23 (0.91, 1.67) 1.23
Garcia (2006) 1.06 (0.93, 1.21) 1.91
Huang (2006) 0.96 (0.80, 1.14) 1.74
Jiao (2011) 1.40 (1.11, 1.76) 1.51
Li (2006) 0.97 (0.81, 1.16) 1.72
Li (2010) 1.02 (0.86, 1.22) 1.73
Liang (2018) 1.02 (0.77, 1.37) 1.28
Liu (2016) 0.92 (0.80, 1.06) 1.88
Long (2010) 0.95 (0.78, 1.16) 1.65
McWilliams (2008) 1.15 (0.94, 1.41) 1.64
Na (2012) 1.22 (0.95, 1.56) 1.43
Santos (2013) 0.90 (0.63, 1.28) 1.05
Zhu (2007) 0.96 (0.79, 1.17) 1.65
Subtotal (I-squared = 7.6%, p = 0.366) 1.04 (0.99, 1.09) 26.69
PB/HB
Ibarrola (2011) 1.01 (0.82, 1.24) 1.62
Sak (2006) 1.23 (1.02, 1.48) 1.69
Subtotal (I-squared = 46.4%, p = 0.172) 1.12 (0.93, 1.35) 3.31
Overall (I-squared = 72.2%, p = 0.000) 1.03 (0.98, 1.09) 100.00
.15
1
6.66

Figure S1

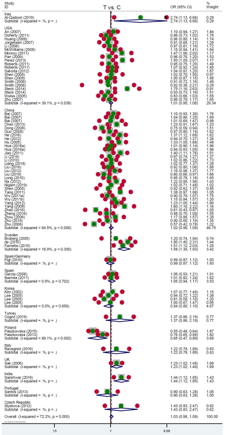
Study
ID
T vs. C
OR (95% CI)
%
Weight
Iraq
Al-Qadoori (2019) 2.74 (1.13, 6.66) 0.29
Subtotal (I-squared = .%, p = .) 2.74 (1.13, 6.66) 0.29
USA
An (2007) 1.10 (0.94, 1.27) 1.84
Doherty (2011) 0.86 (0.73, 1.02) 1.78
Huang (2006) 0.96 (0.80, 1.14) 1.74
Jorgensen (2007) 0.91 (0.68, 1.21) 1.29
Li (2006) 0.97 (0.81, 1.16) 1.72
McWilliams (2008) 1.15 (0.94, 1.41) 1.64
Monroy (2011) 1.47 (1.06, 2.02) 1.17
Pan (2009) 0.96 (0.76, 1.20) 1.53
Perez (2013) 1.50 (1.09, 2.07) 1.17
Roberts (2011) 0.95 (0.75, 1.20) 1.49
Roberts (2011) 1.07 (0.92, 1.24) 1.85
Sakoda (2012) 1.04 (0.91, 1.20) 1.87
Shen (2006) 1.02 (0.70, 1.50) 0.97
Shen (2008) 1.00 (0.87, 1.15) 1.88
Smith (2008) 0.91 (0.72, 1.16) 1.49
Smith (2008) 0.92 (0.38, 2.21) 0.29
Steck (2014) 1.75 (1.16, 2.63) 0.91
Steck (2014) 0.93 (0.73, 1.16) 1.51
Weiss (2005) 0.83 (0.66, 1.03) 1.55
Zhu (2007) 0.96 (0.79, 1.17) 1.65
Subtotal (I-squared = 39.1%, p = 0.038) 1.01 (0.95, 1.08) 29.34
China
Bai (2007) 1.10 (0.93, 1.30) 1.76
Bai (2007) 1.04 (0.86, 1.25) 1.69
Bai (2007) 1.01 (0.69, 1.47) 0.98
Chen (2013) 1.23 (0.91, 1.67) 1.23
Dong (2008) 0.75 (0.59, 0.94) 1.51
Guo (2008) 0.97 (0.80, 1.19) 1.62
He (2016) 1.37 (1.12, 1.68) 1.62
He (2012) 0.94 (0.70, 1.27) 1.26
Hu (2005) 1.33 (1.05, 1.68) 1.49
Hua (2016a) 1.01 (0.90, 1.14) 1.96
Hua (2016a) 0.94 (0.83, 1.05) 1.96
Jiao (2011) 1.40 (1.11, 1.76) 1.51
Li (2014) 0.97 (0.74, 1.27) 1.37
Li (2010) 1.02 (0.86, 1.22) 1.73
Liang (2018) 1.02 (0.77, 1.37) 1.28
Liu (2016) 0.92 (0.80, 1.06) 1.88
Liu (2012) 1.16 (0.98, 1.37) 1.77
Liu (2019) 0.80 (0.66, 0.97) 1.66
Long (2010) 0.95 (0.78, 1.16) 1.65
Na (2012) 1.22 (0.95, 1.56) 1.43
Nigam (2019) 1.02 (0.71, 1.48) 1.02
Shen (2005) 0.92 (0.62, 1.37) 0.95
Tang (2011) 1.38 (0.97, 1.98) 1.05
Wu (2011a) 0.90 (0.76, 1.07) 1.75
Wu (2011b) 1.15 (0.84, 1.57) 1.20
Yang (2012) 1.23 (1.05, 1.44) 1.80
Yang (2008) 1.60 (1.16, 2.22) 1.16
Zhao (2018) 0.61 (0.43, 0.87) 1.05
Zheng (2016) 0.88 (0.70, 1.09) 1.55
Zhou (2008) 1.17 (0.88, 1.57) 1.28
Zhu (2018) 0.90 (0.69, 1.19) 1.35
Zhu (2008) 0.57 (0.42, 0.78) 1.22
Subtotal (I-squared = 64.5%, p = 0.000) 1.02 (0.96, 1.09) 46.75
Sweden
Broberg (2005) 1.20 (0.74, 1.94) 0.74
de (2010) 1.80 (1.40, 2.31) 1.44
Farnebo (2015) 1.51 (1.12, 2.03) 1.25
Subtotal (I-squared = 16.9%, p = 0.300) 1.58 (1.30, 1.93) 3.42
Spain/Germany
Figl (2010) 0.99 (0.87, 1.12) 1.93
Subtotal (I-squared = .%, p = .) 0.99 (0.87, 1.12) 1.93
Spain
Garcia (2006) 1.06 (0.93, 1.21) 1.91
Ibarrola (2011) 1.01 (0.82, 1.24) 1.62
Subtotal (I-squared = 0.0%, p = 0.702) 1.05 (0.94, 1.17) 3.53
Korea
Kim (2002) 1.07 (0.77, 1.49) 1.15
Lee (2005) 0.94 (0.72, 1.22) 1.38
Lee (2005) 0.81 (0.59, 1.10) 1.20
Lee (2005) 1.00 (0.67, 1.47) 0.95
Subtotal (I-squared = 0.0%, p = 0.656) 0.94 (0.80, 1.10) 4.67
Turkey
Ozgoz (2019) 1.37 (0.86, 2.19) 0.77
Subtotal (I-squared = .%, p = .) 1.37 (0.86, 2.19) 0.77
Poland
Paszkowska (2015) 0.55 (0.48, 0.64) 1.87
Paszkowska (2013) 0.76 (0.65, 0.89) 1.82
Subtotal (I-squared = 89.1%, p = 0.002) 0.65 (0.47, 0.89) 3.69
Italy
Ravegnini (2016) 1.22 (0.78, 1.89) 0.83
Subtotal (I-squared = .%, p = .) 1.22 (0.78, 1.89) 0.83
UK
Sak (2006) 1.23 (1.02, 1.48) 1.69
Subtotal (I-squared = .%, p = .) 1.23 (1.02, 1.48) 1.69
India
Sankhwar (2016) 1.44 (1.12, 1.85) 1.43
Subtotal (I-squared = .%, p = .) 1.44 (1.12, 1.85) 1.43
Portugal
Santos (2013) 0.90 (0.63, 1.28) 1.05
Subtotal (I-squared = .%, p = .) 0.90 (0.63, 1.28) 1.05
Czech Republic
Slyskova (2012) 1.43 (0.83, 2.47) 0.62
Subtotal (I-squared = .%, p = .) 1.43 (0.83, 2.47) 0.62
Overall (I-squared = 72.2%, p = 0.000) 1.03 (0.98, 1.09) 100.00
.15
1
6.66

Figure S2

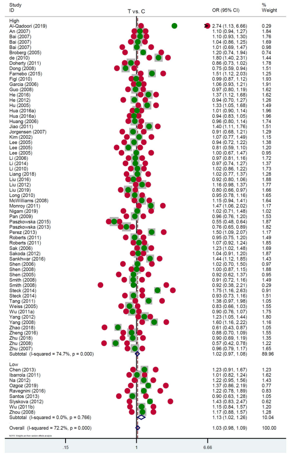

Study
ID
T vs. C
OR (95% CI)
%
Weight
High
Al-Qadoori (2019) 2.74 (1.13, 6.66) 0.29
An (2007) 1.10 (0.94, 1.27) 1.84
Bai (2007) 1.10 (0.93, 1.30) 1.76
Bai (2007) 1.04 (0.86, 1.25) 1.69
Bai (2007) 1.01 (0.69, 1.47) 0.98
Broberg (2005) 1.20 (0.74, 1.94) 0.74
de (2010) 1.80 (1.40, 2.31) 1.44
Doherty (2011) 0.86 (0.73, 1.02) 1.78
Dong (2008) 0.75 (0.59, 0.94) 1.51
Farnebo (2015) 1.51 (1.12, 2.03) 1.25
Figl (2010) 0.99 (0.87, 1.12) 1.93
Garcia (2006) 1.06 (0.93, 1.21) 1.91
Guo (2008) 0.97 (0.80, 1.19) 1.62
He (2016) 1.37 (1.12, 1.68) 1.62
He (2012) 0.94 (0.70, 1.27) 1.26
Hu (2005) 1.33 (1.05, 1.68) 1.49
Hua (2016a) 1.01 (0.90, 1.14) 1.96
Hua (2016a) 0.94 (0.83, 1.05) 1.96
Huang (2006) 0.96 (0.80, 1.14) 1.74
Jiao (2011) 1.40 (1.11, 1.76) 1.51
Jorgensen (2007) 0.91 (0.68, 1.21) 1.29
Kim (2002) 1.07 (0.77, 1.49) 1.15
Lee (2005) 0.94 (0.72, 1.22) 1.38
Lee (2005) 0.81 (0.59, 1.10) 1.20
Lee (2005) 1.00 (0.67, 1.47) 0.95
Li (2006) 0.97 (0.81, 1.16) 1.72
Li (2014) 0.97 (0.74, 1.27) 1.37
Li (2010) 1.02 (0.86, 1.22) 1.73
Liang (2018) 1.02 (0.77, 1.37) 1.28
Liu (2016) 0.92 (0.80, 1.06) 1.88
Liu (2012) 1.16 (0.98, 1.37) 1.77
Liu (2019) 0.80 (0.66, 0.97) 1.66
Long (2010) 0.95 (0.78, 1.16) 1.65
McWilliams (2008) 1.15 (0.94, 1.41) 1.64
Monroy (2011) 1.47 (1.06, 2.02) 1.17
Nigam (2019) 1.02 (0.71, 1.48) 1.02
Pan (2009) 0.96 (0.76, 1.20) 1.53
Paszkowska (2015) 0.55 (0.48, 0.64) 1.87
Paszkowska (2013) 0.76 (0.65, 0.89) 1.82
Perez (2013) 1.50 (1.09, 2.07) 1.17
Roberts (2011) 0.95 (0.75, 1.20) 1.49
Roberts (2011) 1.07 (0.92, 1.24) 1.85
Sak (2006) 1.23 (1.02, 1.48) 1.69
Sakoda (2012) 1.04 (0.91, 1.20) 1.87
Sankhwar (2016) 1.44 (1.12, 1.85) 1.43
Shen (2006) 1.02 (0.70, 1.50) 0.97
Shen (2008) 1.00 (0.87, 1.15) 1.88
Shen (2005) 0.92 (0.62, 1.37) 0.95
Smith (2008) 0.91 (0.72, 1.16) 1.49
Smith (2008) 0.92 (0.38, 2.21) 0.29
Steck (2014) 1.75 (1.16, 2.63) 0.91
Steck (2014) 0.93 (0.73, 1.16) 1.51
Tang (2011) 1.38 (0.97, 1.98) 1.05
Weiss (2005) 0.83 (0.66, 1.03) 1.55
Wu (2011a) 0.90 (0.76, 1.07) 1.75
Yang (2012) 1.23 (1.05, 1.44) 1.80
Yang (2008) 1.60 (1.16, 2.22) 1.16
Zhao (2018) 0.61 (0.43, 0.87) 1.05
Zheng (2016) 0.88 (0.70, 1.09) 1.55
Zhu (2018) 0.90 (0.69, 1.19) 1.35
Zhu (2008) 0.57 (0.42, 0.78) 1.22
Zhu (2007) 0.96 (0.79, 1.17) 1.65
Subtotal (I-squared = 74.7%, p = 0.000) 1.02 (0.97, 1.08) 89.96
Low
Chen (2013) 1.23 (0.91, 1.67) 1.23
Ibarrola (2011) 1.01 (0.82, 1.24) 1.62
Na (2012) 1.22 (0.95, 1.56) 1.43
Ozgoz (2019) 1.37 (0.86, 2.19) 0.77
Ravegnini (2016) 1.22 (0.78, 1.89) 0.83
Santos (2013) 0.90 (0.63, 1.28) 1.05
Slyskova (2012) 1.43 (0.83, 2.47) 0.62
Wu (2011b) 1.15 (0.84, 1.57) 1.20
Zhou (2008) 1.17 (0.88, 1.57) 1.28
Subtotal (I-squared = 0.0%, p = 0.766) 1.13 (1.02, 1.26) 10.04
Overall (I-squared = 72.2%, p = 0.000) 1.03 (0.98, 1.09) 100.00
NOTE: Weights are from random effects analysis
.15
1
6.66

Figure S3

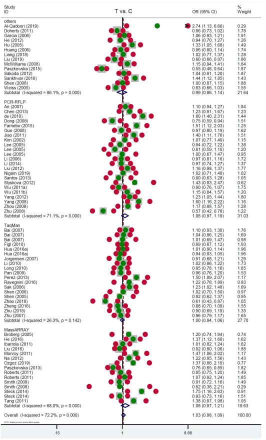
Study
ID
T vs. C
OR (95% CI)
%
Weight
others
Al-Qadoori (2019) 2.74 (1.13, 6.66) 0.29
Doherty (2011) 0.86 (0.73, 1.02) 1.78
Garcia (2006) 1.06 (0.93, 1.21) 1.91
He (2012) 0.94 (0.70, 1.27) 1.26
Hu (2005) 1.33 (1.05, 1.68) 1.49
Huang (2006) 0.96 (0.80, 1.14) 1.74
Liang (2018) 1.02 (0.77, 1.37) 1.28
Liu (2019) 0.80 (0.66, 0.97) 1.66
McWilliams (2008) 1.15 (0.94, 1.41) 1.64
Paszkowska (2015) 0.55 (0.48, 0.64) 1.87
Sakoda (2012) 1.04 (0.91, 1.20) 1.87
Sankhwar (2016) 1.44 (1.12, 1.85) 1.43
Shen (2008) 1.00 (0.87, 1.15) 1.88
Weiss (2005) 0.83 (0.66, 1.03) 1.55
Subtotal (I-squared = 86.1%, p = 0.000) 0.99 (0.86, 1.14) 21.64
PCR-RFLP
An (2007) 1.10 (0.94, 1.27) 1.84
Chen (2013) 1.23 (0.91, 1.67) 1.23
de (2010) 1.80 (1.40, 2.31) 1.44
Dong (2008) 0.75 (0.59, 0.94) 1.51
Farnebo (2015) 1.51 (1.12, 2.03) 1.25
Guo (2008) 0.97 (0.80, 1.19) 1.62
Jiao (2011) 1.40 (1.11, 1.76) 1.51
Kim (2002) 1.07 (0.77, 1.49) 1.15
Lee (2005) 0.94 (0.72, 1.22) 1.38
Lee (2005) 0.81 (0.59, 1.10) 1.20
Lee (2005) 1.00 (0.67, 1.47) 0.95
Li (2006) 0.97 (0.81, 1.16) 1.72
Li (2014) 0.97 (0.74, 1.27) 1.37
Liu (2012) 1.16 (0.98, 1.37) 1.77
Nigam (2019) 1.02 (0.71, 1.48) 1.02
Santos (2013) 0.90 (0.63, 1.28) 1.05
Slyskova (2012) 1.43 (0.83, 2.47) 0.62
Wu (2011a) 0.90 (0.76, 1.07) 1.75
Wu (2011b) 1.15 (0.84, 1.57) 1.20
Yang (2012) 1.23 (1.05, 1.44) 1.80
Yang (2008) 1.60 (1.16, 2.22) 1.16
Zhou (2008) 1.17 (0.88, 1.57) 1.28
Zhu (2008) 0.57 (0.42, 0.78) 1.22
Subtotal (I-squared = 71.1%, p = 0.000) 1.08 (0.97, 1.19) 31.03
TaqMan
Bai (2007) 1.10 (0.93, 1.30) 1.76
Bai (2007) 1.04 (0.86, 1.25) 1.69
Bai (2007) 1.01 (0.69, 1.47) 0.98
Figl (2010) 0.99 (0.87, 1.12) 1.93
Hua (2016a) 1.01 (0.90, 1.14) 1.96
Hua (2016a) 0.94 (0.83, 1.05) 1.96
Jorgensen (2007) 0.91 (0.68, 1.21) 1.29
Li (2010) 1.02 (0.86, 1.22) 1.73
Long (2010) 0.95 (0.78, 1.16) 1.65
Pan (2009) 0.96 (0.76, 1.20) 1.53
Perez (2013) 1.50 (1.09, 2.07) 1.17
Ravegnini (2016) 1.22 (0.78, 1.89) 0.83
Sak (2006) 1.23 (1.02, 1.48) 1.69
Shen (2006) 1.02 (0.70, 1.50) 0.97
Shen (2005) 0.92 (0.62, 1.37) 0.95
Zhao (2018) 0.61 (0.43, 0.87) 1.05
Zheng (2016) 0.88 (0.70, 1.09) 1.55
Zhu (2018) 0.90 (0.69, 1.19) 1.35
Zhu (2007) 0.96 (0.79, 1.17) 1.65
Subtotal (I-squared = 26.3%, p = 0.142) 1.00 (0.94, 1.06) 27.70
MassARRAY
Broberg (2005) 1.20 (0.74, 1.94) 0.74
He (2016) 1.37 (1.12, 1.68) 1.62
Ibarrola (2011) 1.01 (0.82, 1.24) 1.62
Liu (2016) 0.92 (0.80, 1.06) 1.88
Monroy (2011) 1.47 (1.06, 2.02) 1.17
Na (2012) 1.22 (0.95, 1.56) 1.43
Ozgoz (2019) 1.37 (0.86, 2.19) 0.77
Paszkowska (2013) 0.76 (0.65, 0.89) 1.82
Roberts (2011) 0.95 (0.75, 1.20) 1.49
Roberts (2011) 1.07 (0.92, 1.24) 1.85
Smith (2008) 0.91 (0.72, 1.16) 1.49
Smith (2008) 0.92 (0.38, 2.21) 0.29
Steck (2014) 1.75 (1.16, 2.63) 0.91
Steck (2014) 0.93 (0.73, 1.16) 1.51
Tang (2011) 1.38 (0.97, 1.98) 1.05
Subtotal (I-squared = 68.0%, p = 0.000) 1.08 (0.97, 1.21) 19.63
Overall (I-squared = 72.2%, p = 0.000) 1.03 (0.98, 1.09) 100.00
.15
1
6.66

Figure S4

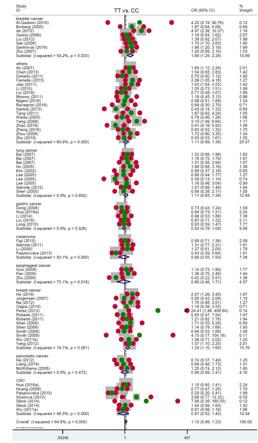
Study
ID
TT vs. CC
OR (95% CI)
%
Weight
bladder cancer
Al-Qadoori (2019) 4.20 (0.19, 90.76) 0.12
Broberg (2005) 2.48 (1.01, 6.09) 0.69
de (2010) 4.97 (2.38, 10.37) 1.19
Garcia (2006) 1.16 (0.84, 1.62) 2.07
Liu (2012) 1.38 (0.92, 2.07) 1.89
Sak (2006) 1.70 (1.10, 2.65) 1.80
Sankhwar (2016) 1.96 (1.20, 3.19) 1.69
Zhu (2007) 1.20 (0.69, 2.10) 1.53
Subtotal (I-squared = 54.2%, p = 0.033) 1.68 (1.25, 2.28) 10.99
others
An (2007) 1.60 (1.12, 2.28) 2.01
Chen (2013) 1.54 (0.83, 2.83) 1.42
Doherty (2011) 0.75 (0.50, 1.12) 1.90
Farnebo (2015) 2.09 (1.05, 4.18) 1.27
Jiao (2011) 1.93 (1.04, 3.55) 1.42
Li (2010) 1.05 (0.73, 1.51) 1.99
Liu (2019) 0.71 (0.48, 1.07) 1.89
Monroy (2011) 1.19 (0.45, 3.13) 0.86
Nigam (2019) 0.98 (0.51, 1.88) 1.34
Ravegnini (2016) 0.89 (0.30, 2.70) 0.71
Santos (2013) 0.43 (0.14, 1.32) 0.69
Tang (2011) 1.87 (0.82, 4.24) 1.05
Weiss (2005) 0.76 (0.46, 1.26) 1.66
Yang (2008) 3.15 (1.49, 6.64) 1.17
Zhao (2018) 0.41 (0.18, 0.92) 1.06
Zheng (2016) 0.83 (0.52, 1.32) 1.75
Zhou (2008) 1.72 (0.90, 3.30) 1.34
Zhu (2018) 0.93 (0.53, 1.61) 1.55
Subtotal (I-squared = 60.9%, p = 0.000) 1.11 (0.89, 1.39) 25.07
lung cancer
Bai (2007) 1.32 (0.89, 1.96) 1.92
Bai (2007) 1.16 (0.75, 1.79) 1.81
Bai (2007) 1.31 (0.58, 2.94) 1.07
Hu (2005) 1.68 (0.88, 3.18) 1.36
Kim (2002) 0.89 (0.37, 2.16) 0.95
Lee (2005) 0.88 (0.44, 1.75) 1.27
Lee (2005) 0.39 (0.13, 1.14) 0.74
Lee (2005) 1.18 (0.46, 3.04) 0.89
Sakoda (2012) 1.01 (0.69, 1.49) 1.94
Shen (2005) 0.89 (0.38, 2.11) 1.00
Subtotal (I-squared = 0.0%, p = 0.602) 1.11 (0.93, 1.34) 12.94
gastric cancer
Dong (2008) 0.73 (0.43, 1.24) 1.59
Hua (2016a) 0.94 (0.73, 1.21) 2.24
Li (2014) 0.99 (0.53, 1.86) 1.38
Liu (2016) 0.97 (0.71, 1.32) 2.11
Long (2010) 0.93 (0.59, 1.47) 1.77
Subtotal (I-squared = 0.0%, p = 0.928) 0.93 (0.78, 1.09) 9.09
melanoma
Figl (2010) 0.99 (0.71, 1.36) 2.09
Ibarrola (2011) 1.31 (0.77, 2.21) 1.61
Li (2008) 1.27 (0.81, 2.00) 1.78
Paszkowska (2013) 0.43 (0.29, 0.64) 1.91
Subtotal (I-squared = 83.1%, p = 0.000) 0.90 (0.55, 1.50) 7.38
esophageal cancer
Guo (2008) 1.14 (0.73, 1.80) 1.77
Pan (2009) 1.36 (0.75, 2.49) 1.44
Zhu (2008) 0.42 (0.22, 0.81) 1.36
Subtotal (I-squared = 75.1%, p = 0.018) 0.89 (0.46, 1.71) 4.57
breast cancer
He (2016) 2.07 (1.26, 3.40) 1.67
Jorgensen (2007) 0.95 (0.43, 2.09) 1.10
Na (2012) 1.76 (0.88, 3.51) 1.27
Ozgoz (2019) 1.18 (0.39, 3.55) 0.71
Perez (2013) 24.41 (1.46, 406.64) 0.14
Roberts (2011) 0.85 (0.47, 1.54) 1.47
Roberts (2011) 1.21 (0.82, 1.78) 1.94
Shen (2006) 1.71 (0.55, 5.28) 0.69
Shen (2008) 1.14 (0.78, 1.66) 1.95
Smith (2008) 0.94 (0.53, 1.68) 1.48
Smith (2008) 4.15 (0.17, 104.16) 0.11
Wu (2011b) 1.46 (0.71, 3.02) 1.20
Yang (2012) 1.57 (1.10, 2.25) 2.01
Subtotal (I-squared = 18.1%, p = 0.261) 1.33 (1.10, 1.60) 15.76
pancreatic cancer
He (2012) 0.74 (0.37, 1.49) 1.25
Liang (2018) 0.89 (0.46, 1.73) 1.31
McWilliams (2008) 1.25 (0.74, 2.12) 1.60
Subtotal (I-squared = 0.0%, p = 0.472) 0.99 (0.69, 1.41) 4.16
CRC
Hua (2016a) 1.10 (0.85, 1.41) 2.24
Huang (2006) 0.77 (0.47, 1.25) 1.70
Paszkowska (2015) 0.29 (0.20, 0.41) 1.99
Slyskova (2012) 3.08 (0.77, 12.32) 0.50
Steck (2014) 7.68 (0.38, 165.05) 0.12
Steck (2014) 1.04 (0.59, 1.83) 1.52
Wu (2011a) 0.81 (0.56, 1.19) 1.96
Subtotal (I-squared = 86.3%, p = 0.000) 0.87 (0.52, 1.45) 10.04
Overall (I-squared = 64.6%, p = 0.000) 1.10 (0.99, 1.23) 100.00
.00246
1
407

Figure S5

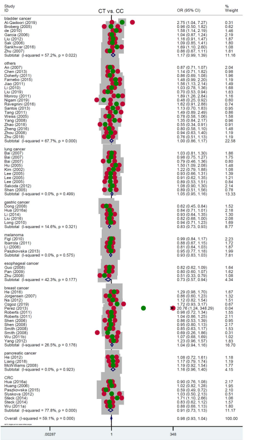

Study
ID
CT vs. CC
OR (95% CI)
%
Weight
bladder cancer
Al-Qadoori (2019) 2.75 (1.04, 7.27) 0.31
Broberg (2005) 0.96 (0.50, 1.82) 0.62
de (2010) 1.58 (1.14, 2.19) 1.46
Garcia (2006) 1.04 (0.87, 1.24) 2.19
Liu (2012) 1.16 (0.91, 1.47) 1.87
Sak (2006) 1.09 (0.85, 1.41) 1.80
Sankhwar (2016) 1.69 (1.10, 2.60) 1.08
Zhu (2007) 0.86 (0.67, 1.11) 1.81
Subtotal (I-squared = 57.2%, p = 0.022) 1.17 (0.99, 1.39) 11.16
others
An (2007) 0.87 (0.71, 1.07) 2.04
Chen (2013) 1.14 (0.71, 1.82) 0.98
Doherty (2011) 0.86 (0.69, 1.08) 1.96
Farnebo (2015) 1.48 (0.99, 2.20) 1.19
Jiao (2011) 1.56 (1.13, 2.14) 1.49
Li (2010) 1.03 (0.78, 1.36) 1.68
Liu (2019) 0.70 (0.53, 0.94) 1.63
Monroy (2011) 1.89 (1.26, 2.84) 1.16
Nigam (2019) 0.48 (0.25, 0.92) 0.60
Ravegnini (2016) 1.62 (0.91, 2.88) 0.74
Santos (2013) 1.13 (0.70, 1.83) 0.95
Tang (2011) 1.49 (0.89, 2.49) 0.86
Weiss (2005) 0.78 (0.58, 1.06) 1.58
Yang (2008) 1.35 (0.84, 2.17) 0.96
Zhao (2018) 0.55 (0.34, 0.91) 0.91
Zheng (2016) 0.80 (0.58, 1.10) 1.48
Zhou (2008) 0.94 (0.63, 1.40) 1.19
Zhu (2018) 0.76 (0.51, 1.13) 1.19
Subtotal (I-squared = 67.7%, p = 0.000) 1.00 (0.86, 1.17) 22.58
lung cancer
Bai (2007) 1.03 (0.81, 1.30) 1.86
Bai (2007) 0.98 (0.75, 1.27) 1.75
Bai (2007) 0.79 (0.46, 1.36) 0.80
Hu (2005) 1.50 (1.09, 2.08) 1.48
Kim (2002) 1.22 (0.79, 1.88) 1.08
Lee (2005) 0.93 (0.66, 1.31) 1.39
Lee (2005) 0.91 (0.62, 1.35) 1.21
Lee (2005) 0.89 (0.53, 1.51) 0.84
Sakoda (2012) 1.08 (0.90, 1.30) 2.14
Shen (2005) 0.89 (0.51, 1.56) 0.78
Subtotal (I-squared = 0.0%, p = 0.499) 1.05 (0.95, 1.16) 13.33
gastric cancer
Dong (2008) 0.62 (0.45, 0.84) 1.52
Hua (2016a) 0.84 (0.71, 1.01) 2.18
Li (2014) 0.93 (0.64, 1.35) 1.30
Liu (2016) 0.82 (0.68, 1.00) 2.08
Long (2010) 0.94 (0.71, 1.23) 1.69
Subtotal (I-squared = 14.6%, p = 0.321) 0.83 (0.73, 0.93) 8.77
melanoma
Figl (2010) 0.99 (0.84, 1.17) 2.23
Ibarrola (2011) 0.88 (0.67, 1.15) 1.72
Li (2006) 0.81 (0.64, 1.03) 1.87
Paszkowska (2013) 0.95 (0.77, 1.18) 1.99
Subtotal (I-squared = 0.0%, p = 0.575) 0.93 (0.83, 1.03) 7.81
esophageal cancer
Guo (2008) 0.82 (0.62, 1.09) 1.64
Pan (2009) 0.80 (0.60, 1.07) 1.62
Zhu (2008) 0.51 (0.33, 0.79) 1.08
Subtotal (I-squared = 42.3%, p = 0.177) 0.73 (0.57, 0.94) 4.34
breast cancer
He (2016) 1.29 (0.98, 1.70) 1.67
Jorgensen (2007) 0.86 (0.60, 1.23) 1.32
Na (2012) 1.12 (0.82, 1.54) 1.51
Ozgoz (2019) 1.72 (0.93, 3.17) 0.67
Perez (2013) 20.76 (1.24, 348.29) 0.04
Roberts (2011) 0.98 (0.72, 1.34) 1.55
Roberts (2011) 1.04 (0.86, 1.25) 2.11
Shen (2006) 0.86 (0.53, 1.39) 0.95
Shen (2008) 0.95 (0.80, 1.13) 2.17
Smith (2008) 0.85 (0.63, 1.17) 1.53
Smith (2008) 0.69 (0.26, 1.86) 0.31
Wu (2011b) 1.07 (0.68, 1.69) 1.02
Yang (2012) 1.23 (0.96, 1.57) 1.83
Subtotal (I-squared = 26.5%, p = 0.176) 1.04 (0.94, 1.16) 16.70
pancreatic cancer
He (2012) 1.08 (0.72, 1.61) 1.18
Liang (2018) 1.17 (0.79, 1.74) 1.19
McWilliams (2008) 1.19 (0.92, 1.54) 1.77
Subtotal (I-squared = 0.0%, p = 0.923) 1.16 (0.96, 1.40) 4.15
CRC
Hua (2016a) 0.90 (0.76, 1.08) 2.17
Huang (2006) 1.02 (0.82, 1.28) 1.95
Paszkowska (2015) 0.59 (0.49, 0.72) 2.10
Slyskova (2012) 1.03 (0.50, 2.13) 0.51
Steck (2014) 1.71 (1.10, 2.66) 1.06
Steck (2014) 0.83 (0.62, 1.12) 1.57
Wu (2011a) 0.88 (0.68, 1.13) 1.80
Subtotal (I-squared = 77.8%, p = 0.000) 0.91 (0.73, 1.13) 11.17
Overall (I-squared = 59.1%, p = 0.000) 0.98 (0.93, 1.04) 100.00
.00287
1
348

Figure S6

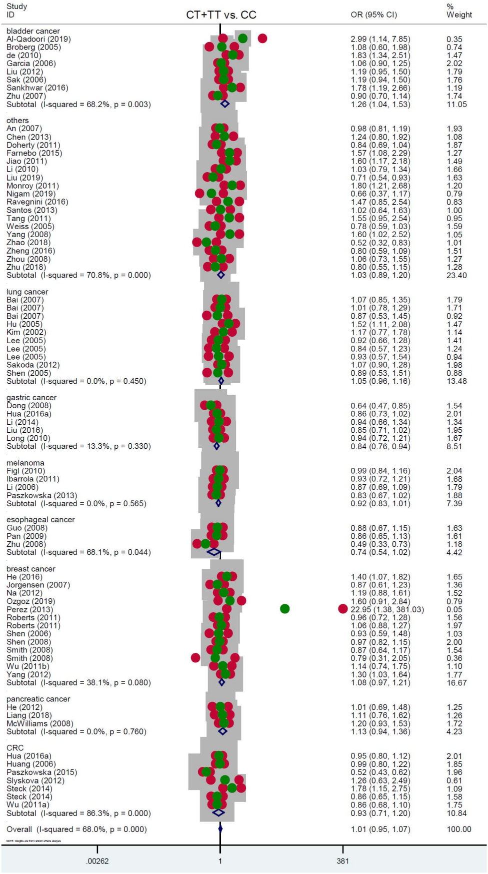
Study ID
CT+TT vs. CC
OR (95% CI)
% Weight
bladder cancer
Al-Qadoori (2019) 2.99 (1.14, 7.85) 0.35
Broberg (2005) 1.08 (0.60, 1.98) 0.74
de (2010) 1.83 (1.34, 2.51) 1.47
Garcia (2006) 1.06 (0.90, 1.25) 2.02
Liu (2012) 1.19 (0.95, 1.50) 1.79
Sak (2006) 1.19 (0.94, 1.50) 1.76
Sankhwar (2016) 1.78 (1.19, 2.66) 1.19
Zhu (2007) 0.90 (0.70, 1.14) 1.74
Subtotal (I-squared = 68.2%, p = 0.003) 1.26 (1.04, 1.53) 11.05
others
An (2007) 0.98 (0.81, 1.19) 1.93
Chen (2013) 1.24 (0.80, 1.92) 1.08
Doherty (2011) 0.84 (0.69, 1.04) 1.87
Farnebo (2015) 1.57 (1.08, 2.29) 1.27
Jiao (2011) 1.60 (1.17, 2.18) 1.49
Li (2010) 1.03 (0.79, 1.34) 1.66
Liu (2019) 0.71 (0.54, 0.93) 1.63
Monroy (2011) 1.80 (1.21, 2.68) 1.20
Nigam (2019) 0.66 (0.37, 1.17) 0.79
Ravegnini (2016) 1.47 (0.85, 2.54) 0.83
Santos (2013) 1.02 (0.64, 1.63) 1.00
Tang (2011) 1.55 (0.95, 2.54) 0.95
Weiss (2005) 0.78 (0.59, 1.03) 1.59
Yang (2008) 1.60 (1.02, 2.52) 1.05
Zhao (2018) 0.52 (0.32, 0.83) 1.01
Zheng (2016) 0.80 (0.59, 1.09) 1.51
Zhou (2008) 1.06 (0.73, 1.55) 1.27
Zhu (2018) 0.80 (0.55, 1.15) 1.28
Subtotal (I-squared = 70.8%, p = 0.000) 1.03 (0.89, 1.20) 23.40
lung cancer
Bai (2007) 1.07 (0.85, 1.35) 1.79
Bai (2007) 1.01 (0.78, 1.29) 1.71
Bai (2007) 0.87 (0.53, 1.45) 0.92
Hu (2005) 1.52 (1.11, 2.08) 1.47
Kim (2002) 1.17 (0.77, 1.78) 1.14
Lee (2005) 0.92 (0.66, 1.28) 1.41
Lee (2005) 0.84 (0.57, 1.23) 1.24
Lee (2005) 0.93 (0.57, 1.54) 0.94
Sakoda (2012) 1.07 (0.90, 1.28) 1.98
Shen (2005) 0.89 (0.53, 1.51) 0.88
Subtotal (I-squared = 0.0%, p = 0.450) 1.05 (0.96, 1.16) 13.48
gastric cancer
Dong (2008) 0.64 (0.47, 0.85) 1.54
Hua (2016a) 0.86 (0.73, 1.02) 2.01
Li (2014) 0.94 (0.66, 1.34) 1.34
Liu (2016) 0.85 (0.71, 1.02) 1.95
Long (2010) 0.94 (0.72, 1.21) 1.67
Subtotal (I-squared = 13.3%, p = 0.330) 0.84 (0.76, 0.94) 8.51
melanoma
Figl (2010) 0.99 (0.84, 1.16) 2.04
Ibarrola (2011) 0.93 (0.72, 1.21) 1.68
Li (2006) 0.87 (0.69, 1.09) 1.79
Paszkowska (2013) 0.83 (0.67, 1.02) 1.88
Subtotal (I-squared = 0.0%, p = 0.565) 0.92 (0.83, 1.01) 7.39
esophageal cancer
Guo (2008) 0.88 (0.67, 1.15) 1.63
Pan (2009) 0.86 (0.65, 1.13) 1.61
Zhu (2008) 0.49 (0.33, 0.73) 1.18
Subtotal (I-squared = 68.1%, p = 0.044) 0.74 (0.54, 1.02) 4.42
breast cancer
He (2016) 1.40 (1.07, 1.82) 1.65
Jorgensen (2007) 0.87 (0.61, 1.23) 1.36
Na (2012) 1.19 (0.88, 1.61) 1.52
Ozgoz (2019) 1.60 (0.91, 2.84) 0.79
Perez (2013) 22.95 (1.38, 381.03) 0.05
Roberts (2011) 0.96 (0.72, 1.28) 1.56
Roberts (2011) 1.06 (0.88, 1.27) 1.97
Shen (2006) 0.93 (0.59, 1.48) 1.03
Shen (2008) 0.97 (0.82, 1.15) 2.00
Smith (2008) 0.87 (0.64, 1.17) 1.54
Smith (2008) 0.79 (0.31, 2.05) 0.36
Wu (2011b) 1.14 (0.74, 1.75) 1.10
Yang (2012) 1.30 (1.03, 1.64) 1.77
Subtotal (I-squared = 38.1%, p = 0.080) 1.08 (0.97, 1.21) 16.67
pancreatic cancer
He (2012) 1.01 (0.69, 1.48) 1.25
Liang (2018) 1.11 (0.76, 1.62) 1.26
McWilliams (2008) 1.20 (0.93, 1.53) 1.72
Subtotal (I-squared = 0.0%, p = 0.760) 1.13 (0.94, 1.36) 4.23
CRC
Hua (2016a) 0.95 (0.80, 1.12) 2.01
Huang (2006) 0.99 (0.80, 1.22) 1.85
Paszkowska (2015) 0.52 (0.43, 0.62) 1.96
Slyskova (2012) 1.26 (0.63, 2.49) 0.61
Steck (2014) 1.78 (1.15, 2.75) 1.09
Steck (2014) 0.86 (0.65, 1.15) 1.58
Wu (2011a) 0.86 (0.68, 1.10) 1.75
Subtotal (I-squared = 86.3%, p = 0.000) 0.93 (0.71, 1.20) 10.84
Overall (I-squared = 68.0%, p = 0.000) 1.01 (0.95, 1.07) 100.00
.00262
1
381

Figure S7

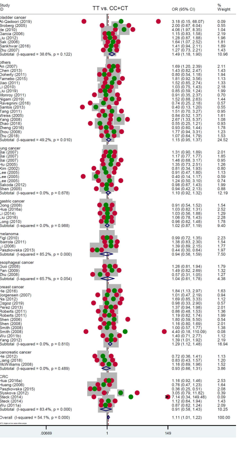
Study ID
TT vs. CC+CT
OR (95% CI)
% Weight
bladder cancer
Al-Qadoori (2019) 3.18 (0.15, 68.07) 0.09
Broberg (2005) 2.00 (0.67, 6.04) 0.55
de (2010) 4.06 (1.97, 8.35) 1.04
Garcia (2006) 1.15 (0.83, 1.58) 2.19
Liu (2012) 1.28 (0.87, 1.88) 1.96
Sak (2006) 1.64 (1.07, 2.52) 1.81
Sankhwar (2016) 1.41 (0.94, 2.11) 1.89
Zhu (2007) 1.27 (0.73, 2.21) 1.43
Subtotal (I-squared = 38.6%, p = 0.122) 1.49 (1.18, 1.90) 10.96
others
An (2007) 1.69 (1.20, 2.39) 2.11
Chen (2013) 1.43 (0.82, 2.47) 1.43
Doherty (2011) 0.80 (0.54, 1.18) 1.94
Farnebo (2015) 1.81 (0.92, 3.56) 1.13
Jiao (2011) 1.52 (0.85, 2.74) 1.33
Li (2010) 1.03 (0.75, 1.43) 2.18
Liu (2019) 0.85 (0.59, 1.24) 1.99
Monroy (2011) 0.91 (0.35, 2.37) 0.70
Nigam (2019) 1.52 (0.88, 2.63) 1.44
Ravegnini (2016) 0.74 (0.25, 2.18) 0.57
Santos (2013) 0.40 (0.13, 1.20) 0.55
Tang (2011) 1.51 (0.70, 3.27) 0.95
Weiss (2005) 0.84 (0.52, 1.37) 1.61
Yang (2008) 2.67 (1.33, 5.37) 1.08
Zhao (2018) 0.55 (0.25, 1.21) 0.93
Zheng (2016) 0.93 (0.60, 1.44) 1.78
Zhou (2008) 1.77 (0.94, 3.31) 1.23
Zhu (2018) 1.07 (0.64, 1.79) 1.53
Subtotal (I-squared = 49.2%, p = 0.010) 1.15 (0.95, 1.37) 24.52
lung cancer
Bai (2007) 1.31 (0.90, 1.89) 2.01
Bai (2007) 1.17 (0.77, 1.77) 1.85
Bai (2007) 1.46 (0.68, 3.17) 0.95
Hu (2005) 1.35 (0.73, 2.51) 1.26
Kim (2002) 0.81 (0.34, 1.93) 0.80
Lee (2005) 0.91 (0.47, 1.80) 1.13
Lee (2005) 0.40 (0.14, 1.17) 0.59
Lee (2005) 1.24 (0.50, 3.10) 0.74
Sakoda (2012) 0.98 (0.67, 1.43) 1.99
Shen (2005) 0.94 (0.42, 2.13) 0.88
Subtotal (I-squared = 0.0%, p = 0.678) 1.10 (0.92, 1.32) 12.19
gastric cancer
Dong (2008) 0.91 (0.54, 1.52) 1.54
Hua (2016a) 1.03 (0.82, 1.31) 2.52
Li (2014) 1.03 (0.56, 1.88) 1.29
Liu (2016) 1.06 (0.78, 1.43) 2.28
Long (2010) 0.96 (0.62, 1.48) 1.78
Subtotal (I-squared = 0.0%, p = 0.988) 1.02 (0.87, 1.19) 9.40
melanoma
Figl (2010) 0.99 (0.72, 1.35) 2.23
Ibarrola (2011) 1.38 (0.83, 2.30) 1.54
Li (2006) 1.39 (0.89, 2.15) 1.77
Paszkowska (2013) 0.44 (0.30, 0.64) 1.97
Subtotal (I-squared = 85.2%, p = 0.000) 0.94 (0.56, 1.59) 7.50
esophageal cancer
Guo (2008) 1.26 (0.81, 1.94) 1.79
Pan (2009) 1.49 (0.82, 2.69) 1.32
Zhu (2008) 0.57 (0.31, 1.05) 1.27
Subtotal (I-squared = 65.7%, p = 0.054) 1.04 (0.61, 1.78) 4.38
breast cancer
He (2016) 1.84 (1.13, 2.97) 1.63
Jorgensen (2007) 1.01 (0.47, 2.19) 0.94
Na (2012) 1.69 (0.85, 3.33) 1.12
Ozgoz (2019) 0.98 (0.33, 2.90) 0.57
Perez (2013) 1.37 (0.94, 1.98) 2.01
Roberts (2011) 0.86 (0.48, 1.53) 1.36
Roberts (2011) 1.19 (0.82, 1.74) 1.99
Shen (2006) 1.80 (0.59, 5.50) 0.54
Shen (2008) 1.16 (0.80, 1.69) 2.01
Smith (2008) 1.00 (0.57, 1.77) 1.38
Smith (2008) 4.40 (0.18, 110.09) 0.08
Wu (2011b) 1.40 (0.71, 2.77) 1.12
Yang (2012) 1.39 (1.01, 1.92) 2.19
Subtotal (I-squared = 0.0%, p = 0.810) 1.29 (1.12, 1.48) 16.94
pancreatic cancer
He (2012) 0.72 (0.36, 1.41) 1.13
Liang (2018) 0.83 (0.43, 1.57) 1.20
McWilliams (2008) 1.16 (0.69, 1.96) 1.52
Subtotal (I-squared = 0.0%, p = 0.489) 0.93 (0.66, 1.31) 3.86
CRC
Hua (2016a) 1.16 (0.92, 1.46) 2.53
Huang (2006) 0.76 (0.47, 1.23) 1.64
Paszkowska (2015) 0.36 (0.25, 0.51) 2.08
Slyskova (2012) 3.05 (0.79, 11.82) 0.39
Steck (2014) 7.14 (0.34, 149.46) 0.09
Steck (2014) 1.12 (0.64, 1.94) 1.43
Wu (2011a) 0.87 (0.62, 1.24) 2.09
Subtotal (I-squared = 83.4%, p = 0.000) 0.91 (0.58, 1.43) 10.25
Overall (I-squared = 54.1%, p = 0.000) 1.11 (1.01, 1.22) 100.00
.00669
1
149

Figure S8

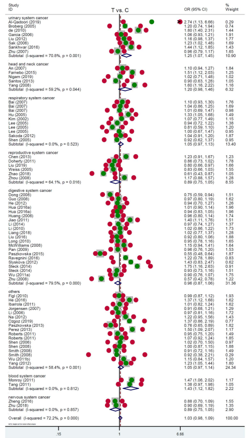

Study
ID
T vs. C
OR (95% CI)
%
Weight
urinary system cancer
Al-Qadoori (2019) 2.74 (1.13, 6.66) 0.29
Broberg (2005) 1.20 (0.74, 1.94) 0.74
de (2010) 1.80 (1.40, 2.31) 1.44
Garcia (2006) 1.06 (0.93, 1.21) 1.91
Liu (2012) 1.16 (0.98, 1.37) 1.77
Sak (2006) 1.23 (1.02, 1.48) 1.69
Sankhwar (2016) 1.44 (1.12, 1.85) 1.43
Zhu (2007) 0.96 (0.79, 1.17) 1.65
Subtotal (I-squared = 70.8%, p = 0.001) 1.25 (1.07, 1.45) 10.90
head and neck cancer
An (2007) 1.10 (0.94, 1.27) 1.84
Farnebo (2015) 1.51 (1.12, 2.03) 1.25
Nigam (2019) 1.02 (0.71, 1.48) 1.02
Santos (2013) 0.90 (0.63, 1.28) 1.05
Yang (2008) 1.60 (1.16, 2.22) 1.16
Subtotal (I-squared = 59.2%, p = 0.044) 1.20 (0.98, 1.46) 6.32
respiratory system cancer
Bai (2007) 1.10 (0.93, 1.30) 1.76
Bai (2007) 1.04 (0.86, 1.25) 1.69
Bai (2007) 1.01 (0.69, 1.47) 0.98
Hu (2005) 1.33 (1.05, 1.68) 1.49
Kim (2002) 1.07 (0.77, 1.49) 1.15
Lee (2005) 0.94 (0.72, 1.22) 1.38
Lee (2005) 0.81 (0.59, 1.10) 1.20
Lee (2005) 1.00 (0.67, 1.47) 0.95
Sakoda (2012) 1.04 (0.91, 1.20) 1.87
Shen (2005) 0.92 (0.62, 1.37) 0.95
Subtotal (I-squared = 0.0%, p = 0.523) 1.05 (0.97, 1.13) 13.40
reproductive system cancer
Chen (2013) 1.23 (0.91, 1.67) 1.23
Doherty (2011) 0.86 (0.73, 1.02) 1.78
Liu (2019) 0.80 (0.66, 0.97) 1.66
Weiss (2005) 0.83 (0.66, 1.03) 1.55
Zhao (2018) 0.61 (0.43, 0.87) 1.05
Zhou (2008) 1.17 (0.88, 1.57) 1.28
Subtotal (I-squared = 64.1%, p = 0.016) 0.89 (0.75, 1.05) 8.55
digestive system cancer
Dong (2008) 0.75 (0.59, 0.94) 1.51
Guo (2008) 0.97 (0.80, 1.19) 1.62
He (2012) 0.94 (0.70, 1.27) 1.26
Hua (2016a) 1.01 (0.90, 1.14) 1.96
Hua (2016a) 0.94 (0.83, 1.05) 1.96
Huang (2006) 0.96 (0.80, 1.14) 1.74
Jiao (2011) 1.40 (1.11, 1.76) 1.51
Li (2014) 0.97 (0.74, 1.27) 1.37
Li (2010) 1.02 (0.86, 1.22) 1.73
Liang (2018) 1.02 (0.77, 1.37) 1.28
Liu (2016) 0.92 (0.80, 1.06) 1.88
Long (2010) 0.95 (0.78, 1.16) 1.65
McWilliams (2008) 1.15 (0.94, 1.41) 1.64
Pan (2009) 0.96 (0.76, 1.20) 1.53
Paszkowska (2015) 0.55 (0.48, 0.64) 1.87
Ravegnini (2016) 1.22 (0.78, 1.89) 0.83
Slyskova (2012) 1.43 (0.83, 2.47) 0.62
Steck (2014) 1.75 (1.16, 2.63) 0.91
Steck (2014) 0.93 (0.73, 1.16) 1.51
Wu (2011a) 0.90 (0.76, 1.07) 1.75
Zhu (2008) 0.57 (0.42, 0.78) 1.22
Subtotal (I-squared = 79.5%, p = 0.000) 0.96 (0.87, 1.06) 31.36
others
Figl (2010) 0.99 (0.87, 1.12) 1.93
He (2016) 1.37 (1.12, 1.68) 1.62
Ibarrola (2011) 1.01 (0.82, 1.24) 1.62
Jorgensen (2007) 0.91 (0.68, 1.21) 1.29
Li (2006) 0.97 (0.81, 1.16) 1.72
Na (2012) 1.22 (0.95, 1.56) 1.43
Ozgoz (2019) 1.37 (0.86, 2.19) 0.77
Paszkowska (2013) 0.76 (0.65, 0.89) 1.82
Perez (2013) 1.50 (1.09, 2.07) 1.17
Roberts (2011) 0.95 (0.75, 1.20) 1.49
Roberts (2011) 1.07 (0.92, 1.24) 1.85
Shen (2006) 1.02 (0.70, 1.50) 0.97
Shen (2008) 1.00 (0.87, 1.15) 1.88
Smith (2008) 0.91 (0.72, 1.16) 1.49
Smith (2008) 0.92 (0.38, 2.21) 0.29
Wu (2011b) 1.15 (0.84, 1.57) 1.20
Yang (2012) 1.23 (1.05, 1.44) 1.80
Subtotal (I-squared = 58.4%, p = 0.001) 1.05 (0.97, 1.14) 24.34
blood system cancer
Monroy (2011) 1.47 (1.06, 2.02) 1.17
Tang (2011) 1.38 (0.97, 1.98) 1.05
Subtotal (I-squared = 0.0%, p = 0.812) 1.43 (1.12, 1.82) 2.22
nervous system cancer
Zheng (2016) 0.88 (0.70, 1.09) 1.55
Zhu (2018) 0.90 (0.69, 1.19) 1.35
Subtotal (I-squared = 0.0%, p = 0.857) 0.89 (0.75, 1.05) 2.90
Overall (I-squared = 72.2%, p = 0.000) 1.03 (0.98, 1.09) 100.00
.15
1
6.66

Figure S9

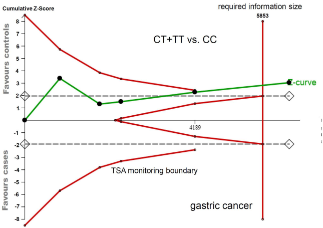
Cumulative Z-Score
required information size
5853
CT+TT vs. CC
Favours controls
Favours cases
Z-curve
4189
TSA monitoring boundary
gastric cancer
8
7
6
5
4
3
2
1.
-1
-2
-3
-4
-5
-6
-7
-8

Figure S10

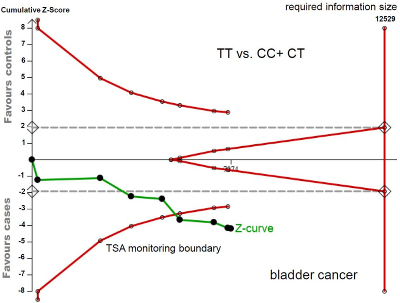
Cumulative Z-Score
required information size
12529
TT vs. CC+ CT
Favours controls
Favours cases
Z-curve
TSA monitoring boundary
bladder cancer

Figure S11

Table S1. Database search strategy in our study.

PubMed

#1 Search (((((((((((((((((Neoplasms) OR Neoplasia) OR Neoplasias) OR Neoplasm) OR Tumors) OR Tumor) OR Cancer) OR Cancers) OR Malignancy) OR Malignancies) OR Malignant Neoplasms) OR Malignant Neoplasm) OR Neoplasm, Malignant) OR Neoplasms, Malignant) OR Benign Neoplasms) OR Neoplasms, Benign) OR Benign Neoplasm) OR Neoplasm, Benign **4,438,109**

#2 Search ((((((((((XPC) OR xeroderma pigmentosum complementation group C) OR Xeroderma Pigmentosum, Complementation Group C) OR (XPC Complex Subunit, DNA Damage Recognition And Repair Factor)) OR DNA Repair Protein Complementing XP-C Cells) OR XPCC) OR P125) OR Xeroderma Pigmentosum Group C-Complementing Protein) OR Mutant Xeroderma Pigmentosum Group C) OR RAD4) OR XP3 **1,662**

#3 #1 and #2

Search (((((((((((((((((((Neoplasms) OR Neoplasia) OR Neoplasias) OR Neoplasm) OR Tumors) OR Tumor) OR Cancer) OR Cancers) OR Malignancy) OR Malignancies) OR Malignant Neoplasms) OR Malignant Neoplasm) OR Neoplasm, Malignant) OR Neoplasms, Malignant) OR Benign Neoplasms) OR Neoplasms, Benign) OR Benign Neoplasm) OR Neoplasm, Benign)) AND (((((((((((XPC) OR xeroderma pigmentosum complementation group C) OR Xeroderma Pigmentosum, Complementation Group C) OR (XPC Complex Subunit, DNA Damage Recognition And Repair Factor)) OR DNA Repair Protein Complementing XP-C Cells) OR XPCC) OR P125) OR Xeroderma Pigmentosum Group C-Complementing Protein) OR Mutant Xeroderma Pigmentosum Group C) OR RAD4) OR XP3) **916**

#4 Search (((((Polymorphism, Genetic) OR Polymorphisms, Genetic) OR Genetic Polymorphisms) OR Genetic Polymorphism) OR Polymorphism (Genetics)) OR Polymorphisms (Genetics) **300,850**

#5 #3 and #4

Search (((((((((((((((((((((Neoplasms) OR Neoplasia) OR Neoplasias) OR Neoplasm) OR Tumors) OR Tumor) OR Cancer) OR Cancers) OR Malignancy) OR Malignancies) OR Malignant Neoplasms) OR Malignant Neoplasm) OR Neoplasm, Malignant) OR Neoplasms, Malignant) OR Benign Neoplasms) OR Neoplasms, Benign) OR Benign Neoplasm) OR Neoplasm, Benign)) AND (((((((((((XPC) OR xeroderma pigmentosum complementation group C) OR Xeroderma Pigmentosum, Complementation Group C) OR (XPC Complex Subunit, DNA Damage Recognition And Repair Factor)) OR DNA Repair Protein Complementing XP-C Cells) OR XPCC) OR P125) OR Xeroderma Pigmentosum Group C-Complementing Protein) OR Mutant Xeroderma Pigmentosum

Group C) OR RAD4) OR XP3))) AND ((((((Polymorphism, Genetic) OR Polymorphisms, Genetic) OR Genetic Polymorphisms) OR Genetic Polymorphism) OR Polymorphism (Genetics)) OR Polymorphisms (Genetics)) **266**

Embase

#1 'xpc' OR 'xeroderma pigmentosum complementation group c' OR 'xeroderma pigmentosum, complementation group c' OR 'xpcc' OR 'p125' OR 'xeroderma pigmentosum group c-complementing protein'/exp OR 'xeroderma pigmentosum group c-complementing protein' OR 'mutant xeroderma pigmentosum group c' OR 'rad4' OR 'xp3' **3,077**

#2 'neoplasm'/exp OR 'neoplasm' OR 'acral tumor'/exp OR 'acral tumor' OR 'acral tumour'/exp OR 'acral tumour' OR 'neoplasia'/exp OR 'neoplasia' OR 'neoplasms'/exp OR 'neoplasms' OR 'neoplasms by histologic type'/exp OR 'neoplasms by histologic type' OR 'neoplasms, cystic, mucinous, and serous'/exp OR 'neoplasms, cystic, mucinous, and serous' OR 'neoplasms, embryonal and mixed'/exp OR 'neoplasms, embryonal and mixed' OR 'neoplasms, germ cell and embryonal'/exp OR 'neoplasms, germ cell and embryonal' OR 'neoplasms, glandular and epithelial'/exp OR 'neoplasms, glandular and epithelial' OR 'neoplasms, hormone-dependent'/exp OR 'neoplasms, hormone-dependent' OR 'neoplasms, post-traumatic'/exp OR 'neoplasms, post-traumatic' OR 'neoplastic disease'/exp OR 'neoplastic disease' OR 'tumor'/exp OR 'tumor' OR 'tumour'/exp OR 'tumour' **5,369,297**

#3 'genetic polymorphism'/exp OR 'genetic polymorphism' OR 'polymorphism (genetics)'/exp OR 'polymorphism (genetics)' OR 'polymorphism, genetic'/exp OR 'polymorphism, genetic' OR 'polymorphism'/exp OR 'polymorphism' OR 'polymorphisms' OR 'mutations'/exp OR 'mutations' OR 'mutation'/exp OR 'mutation' **1,699,261**

#4 #1 and #2 and #3 **687**

CNKI

(keyword= Polymorphisms) And (keyword= XPC) **28**

WOS

#1 TOPIC: (Neoplasms) OR TOPIC: (Neoplasia) OR TOPIC: (Neoplasias) OR TOPIC: (Neoplasm) OR TOPIC: (Tumors) OR TOPIC: (Tumor) OR TOPIC: (Cancer) OR TOPIC: (Cancers) **3,784,624**

#2 TOPIC: (XPC) OR TOPIC: (xeroderma pigmentosum complementation group C) OR TOPIC: (XPC Complex Subunit, DNA Damage Recognition And Repair Factor) OR TOPIC: (XPCC) OR TOPIC: (P125) OR TOPIC: (Mutant Xeroderma Pigmentosum Group C) OR TOPIC: (RAD4) OR TOPIC: (XP3) **2,435**

#3 TOPIC: (Polymorphism, Genetic) OR TOPIC: (Polymorphisms, Genetic) OR TOPIC: (Genetic Polymorphisms) OR TOPIC: (Genetic Polymorphism) ORTOPIC: (Polymorphism (Genetics)) OR TOPIC: (Polymorphisms (Genetics)) **394,549**

**#4**	#1 and #2 and #3	**205**

CNKI, China National Knowledge Infrastructure; WOS, Web of Science.

Table S2. Article quality assessment using the NOS system.

First author	Year	Score	Case		Control		Comparability		Exposure	Method	Non-response
			Definition	Representativeness	Selection	Definition	Factor 1	Factor 1			
**Al-Qadoori**	**2019**	**8**	*1*	*1*	*1*	*1*	*1*	*1*	*0*	*1*	*1*
**An**	**2007**	**8**	*1*	*1*	*0*	*1*	*1*	*1*	*1*	*1*	*1*
**Bai**	**2007**	**7**	*1*	*1*	*0*	*1*	*1*	*1*	*1*	*1*	*0*
**Broberg**	**2005**	**8**	*1*	*1*	*1*	*1*	*1*	*1*	*1*	*1*	*0*
**Chen**	**2013**	**7**	*1*	*1*	*0*	*0*	*1*	*1*	*1*	*1*	*1*
**de**	**2010**	**6**	*1*	*1*	*1*	*1*	*1*	*0*	*0*	*1*	*0*
**Doherty**	**2011**	**8**	*1*	*1*	*1*	*1*	*1*	*1*	*1*	*1*	*0*
**Dong**	**2008**	**9**	*1*	*1*	*1*	*1*	*1*	*1*	*1*	*1*	*1*
**Farnebo**	**2015**	**9**	*1*	*1*	*1*	*1*	*1*	*1*	*1*	*1*	*1*
**Figl**	**2010**	**6**	*1*	*1*	*1*	*1*	*0*	*1*	*0*	*1*	*0*
**Garcia**	**2006**	**6**	*1*	*1*	*0*	*0*	*1*	*1*	*1*	*1*	*0*
**Guo**	**2008**	**9**	*1*	*1*	*1*	*1*	*1*	*1*	*1*	*1*	*1*
**He**	**2016**	**9**	*1*	*1*	*1*	*1*	*1*	*1*	*1*	*1*	*1*
**He**	**2012**	**8**	*1*	*1*	*1*	*1*	*1*	*1*	*1*	*1*	*0*
**Hu**	**2005**	**8**	*1*	*1*	*1*	*1*	*1*	*1*	*1*	*1*	*0*
**Hua**	**2016a**	**9**	*1*	*1*	*1*	*1*	*1*	*1*	*1*	*1*	*1*
**Hua**	**2016b**	**9**	*1*	*1*	*1*	*1*	*1*	*1*	*1*	*1*	*1*
**Huang**	**2006**	**6**	*1*	*1*	*0*	*0*	*1*	*1*	*1*	*1*	*0*
**Ibarrola**	**2011**	**6**	*1*	*1*	*0*	*1*	*0*	*1*	*1*	*1*	*0*
**Jiao**	**2011**	**7**	*1*	*1*	*0*	*1*	*1*	*1*	*0*	*1*	*1*

**Jorgensen**	**2007**	**7**	*1*	*1*	*1*	*1*	*1*	*1*	*0*	*1*	*0*
**Kim**	**2002**	**8**	*1*	*1*	*1*	*1*	*1*	*1*	*1*	*1*	*0*
**Lee**	**2005**	**9**	*1*	*1*	*1*	*1*	*1*	*1*	*1*	*1*	*1*
**Li**	**2006**	**6**	*1*	*1*	*0*	*0*	*1*	*1*	*0*	*1*	*1*
**Li**	**2014**	**8**	*1*	*1*	*1*	*1*	*1*	*1*	*0*	*1*	*1*
**Li**	**2010**	**7**	*1*	*1*	*0*	*0*	*1*	*1*	*1*	*1*	*1*
**Liang**	**2018**	**7**	*1*	*1*	*0*	*0*	*1*	*1*	*1*	*1*	*1*
**Liu**	**2016**	**6**	*1*	*1*	*0*	*1*	*0*	*1*	*1*	*1*	*0*
**Liu**	**2012**	**9**	*1*	*1*	*1*	*1*	*1*	*1*	*1*	*1*	*1*
**Liu**	**2019**	**8**	*1*	*1*	*1*	*1*	*1*	*0*	*1*	*1*	*1*
**Long**	**2010**	**7**	*1*	*1*	*0*	*0*	*1*	*1*	*1*	*1*	*1*
**McWilliams**	**2008**	**6**	*1*	*1*	*0*	*1*	*0*	*1*	*1*	*1*	*0*
**Monroy**	**2011**	**9**	*1*	*1*	*1*	*1*	*1*	*1*	*1*	*1*	*1*
**Na**	**2012**	**7**	*1*	*1*	*0*	*0*	*1*	*1*	*1*	*1*	*1*
**Nigam**	**2019**	**7**	*1*	*1*	*1*	*1*	*1*	*1*	*0*	*1*	*0*
**Ozgoz**	**2019**	**8**	*1*	*1*	*1*	*1*	*0*	*1*	*1*	*1*	*1*
**Pan**	**2009**	**8**	*1*	*1*	*1*	*1*	*1*	*1*	*1*	*1*	*0*
**Paszkowska**	**2015**	**6**	*1*	*1*	*1*	*1*	*0*	*1*	*0*	*1*	*0*
**Paszkowska**	**2013**	**6**	*1*	*1*	*1*	*1*	*0*	*1*	*0*	*1*	*0*
**Perez**	**2013**	**8**	*1*	*1*	*1*	*1*	*1*	*1*	*1*	*1*	*0*
**Ravegnini**	**2016**	**8**	*1*	*1*	*1*	*1*	*1*	*1*	*0*	*1*	*1*
**Roberts**	**2011**	**8**	*1*	*1*	*1*	*1*	*1*	*1*	*1*	*1*	*0*
**Sak**	**2006**	**6**	*1*	*1*	*0*	*1*	*1*	*0*	*1*	*1*	*0*
**Sakoda**	**2012**	**8**	*1*	*1*	*1*	*1*	*1*	*1*	*1*	*1*	*0*

**Sankhwar**	**2016**	**8**	*1*	*1*	*1*	*1*	*0*	*1*	*1*	*1*	*1*
**Santos**	**2013**	**8**	*1*	*1*	*0*	*1*	*1*	*1*	*1*	*1*	*1*
**Shen**	**2006**	**7**	*1*	*1*	*1*	*1*	*0*	*1*	*1*	*1*	*0*
**Shen**	**2008**	**7**	*1*	*1*	*1*	*1*	*1*	*0*	*1*	*1*	*0*
**Shen**	**2005**	**8**	*1*	*1*	*1*	*1*	*1*	*1*	*1*	*1*	*0*
**Slyskova**	**2012**	**8**	*1*	*1*	*1*	*1*	*1*	*1*	*1*	*1*	*0*
**Smith**	**2008**	**8**	*1*	*1*	*1*	*1*	*1*	*1*	*1*	*1*	*0*
**Steck**	**2014**	**8**	*1*	*1*	*1*	*1*	*1*	*1*	*1*	*1*	*0*
**Tang**	**2011**	**7**	*1*	*1*	*1*	*1*	*1*	*1*	*0*	*1*	*0*
**Weiss**	**2005**	**9**	*1*	*1*	*1*	*1*	*1*	*1*	*1*	*1*	*1*
**Wu**	**2011a**	**8**	*1*	*1*	*1*	*1*	*1*	*1*	*1*	*1*	*0*
**Wu**	**2011b**	**8**	*1*	*1*	*1*	*1*	*1*	*1*	*1*	*1*	*0*
**Yang**	**2012**	**8**	*1*	*1*	*1*	*1*	*1*	*1*	*0*	*1*	*1*
**Yang**	**2008**	**9**	*1*	*1*	*1*	*1*	*1*	*1*	*1*	*1*	*1*
**Zhao**	**2018**	**8**	*1*	*1*	*1*	*1*	*1*	*1*	*0*	*1*	*1*
**Zheng**	**2016**	**7**	*1*	*1*	*1*	*1*	*1*	*1*	*0*	*1*	*0*
**Zhou**	**2008**	**8**	*1*	*1*	*1*	*1*	*1*	*1*	*0*	*1*	*1*
**Zhu**	**2018**	**8**	*1*	*1*	*1*	*1*	*1*	*1*	*0*	*1*	*1*
**Zhu**	**2008**	**8**	*1*	*1*	*1*	*1*	*1*	*1*	*0*	*1*	*1*
**Zhu**	**2007**	**6**	*1*	*1*	*0*	*0*	*1*	*1*	*1*	*1*	*0*

NOS, Newcastle-Ottawa quality assessment Scale.

Table S3. Article quality assessment using the risk-of-bias score system.

First author	Year	Score	Representativeness of case[a]	Representativeness of control[b]	Ascertainment of case[c]	Control selection[d]	Genotyping examination[e]	HWE[f]	Total sample size[g]
**Al-Qadoori**	**2019**	**10**	*1*	*3*	*2*	*2*	*1*	*1*	*0*
**An**	**2007**	**11**	*1*	*1*	*2*	*2*	*1*	*1*	*3*
**Bai**	**2007**	**11**	*1*	*1*	*2*	*2*	*2*	*1*	*2*
**Broberg**	**2005**	**11**	*1*	*3*	*2*	*2*	*1*	*1*	*1*
**Chen**	**2013**	**9**	*1*	*1*	*2*	*2*	*1*	*1*	*1*
**de**	**2010**	**10**	*2*	*3*	*1*	*1*	*0*	*1*	*2*
**Doherty**	**2011**	**13**	*2*	*3*	*1*	*1*	*2*	*1*	*3*
**Dong**	**2008**	**13**	*2*	*3*	*2*	*2*	*1*	*1*	*2*
**Farnebo**	**2015**	**10**	*1*	*3*	*0*	*2*	*1*	*1*	*2*
**Figl**	**2010**	**11**	*1*	*3*	*2*	*1*	*0*	*1*	*3*
**Garcia**	**2006**	**12**	*2*	*1*	*2*	*2*	*1*	*1*	*3*
**Guo**	**2008**	**10**	*2*	*3*	*1*	*1*	*0*	*1*	*2*
**He**	**2016**	**10**	*1*	*3*	*2*	*1*	*0*	*1*	*2*
**He**	**2012**	**10**	*1*	*3*	*2*	*2*	*0*	*1*	*1*
**Hu**	**2005**	**12**	*1*	*3*	*2*	*2*	*1*	*1*	*2*
**Hua**	**2016a**	**13**	*1*	*3*	*2*	*1*	*2*	*1*	*3*
**Hua**	**2016b**	**11**	*1*	*3*	*2*	*1*	*0*	*1*	*3*
**Huang**	**2006**	**10**	*2*	*1*	*0*	*1*	*2*	*1*	*3*
**Ibarrola**	**2011**	**8**	*1*	*3*	*0*	*0*	*1*	*1*	*2*
**Jiao**	**2011**	**10**	*1*	*1*	*2*	*2*	*1*	*1*	*2*

**Jorgensen**	**2007**	**11**	*2*	*3*	*1*	*2*	*0*	*1*	*2*
**Kim**	**2002**	**10**	*1*	*3*	*2*	*2*	*0*	*1*	*1*
**Lee**	**2005**	**12**	*1*	*3*	*2*	*2*	*1*	*1*	*2*
**Li**	**2006**	**12**	*2*	*1*	*2*	*2*	*1*	*1*	*3*
**Li**	**2014**	**12**	*1*	*3*	*2*	*2*	*1*	*1*	*2*
**Li**	**2010**	**12**	*1*	*1*	*2*	*2*	*2*	*1*	*3*
**Liang**	**2018**	**10**	*1*	*1*	*2*	*2*	*2*	*1*	*1*
**Liu**	**2016**	**10**	*2*	*1*	*2*	*0*	*1*	*1*	*3*
**Liu**	**2012**	**13**	*1*	*3*	*2*	*2*	*1*	*1*	*3*
**Liu**	**2019**	**11**	*1*	*3*	*2*	*1*	*1*	*1*	*2*
**Long**	**2010**	**10**	*1*	*1*	*2*	*2*	*1*	*1*	*2*
**McWilliams**	**2008**	**11**	*2*	*1*	*2*	*0*	*2*	*1*	*3*
**Monroy**	**2011**	**12**	*2*	*3*	*2*	*2*	*1*	*1*	*1*
**Na**	**2012**	**8**	*1*	*1*	*2*	*1*	*1*	*1*	*1*
**Nigam**	**2019**	**10**	*2*	*3*	*1*	*1*	*0*	*1*	*2*
**Ozgoz**	**2019**	**8**	*1*	*3*	*2*	*0*	*0*	*1*	*1*
**Pan**	**2009**	**13**	*2*	*3*	*1*	*2*	*2*	*1*	*2*
**Paszkowska**	**2015**	**10**	*2*	*3*	*1*	*0*	*0*	*1*	*3*
**Paszkowska**	**2013**	**10**	*2*	*3*	*1*	*0*	*0*	*1*	*3*
**Perez**	**2013**	**10**	*2*	*3*	*2*	*0*	*0*	*1*	*2*
**Ravegnini**	**2016**	**7**	*2*	*2*	*0*	*0*	*1*	*1*	*1*
**Roberts**	**2011**	**13**	*1*	*3*	*2*	*2*	*1*	*1*	*3*
**Sak**	**2006**	**10**	*1*	*0*	*2*	*2*	*1*	*1*	*3*
**Sakoda**	**2012**	**13**	*1*	*3*	*1*	*2*	*2*	*1*	*3*
**Sankhwar**	**2016**	**10**	*1*	*3*	*2*	*0*	*1*	*1*	*2*
**Santos**	**2013**	**9**	*1*	*1*	*2*	*2*	*1*	*1*	*1*

**Shen**	**2006**	**10**	*2*	*3*	*1*	*1*	*1*	*1*	*1*
**Shen**	**2008**	**13**	*2*	*3*	*1*	*1*	*2*	*1*	*3*
**Shen**	**2005**	**10**	*1*	*3*	*1*	*2*	*1*	*1*	*1*
**Slyskova**	**2012**	**9**	*1*	*3*	*2*	*1*	*1*	*1*	*0*
**Smith**	**2008**	**12**	*2*	*3*	*2*	*1*	*1*	*1*	*2*
**Steck**	**2014**	**10**	*2*	*1*	*1*	*2*	*1*	*1*	*2*
**Tang**	**2011**	**10**	*1*	*3*	*2*	*2*	*0*	*1*	*1*
**Weiss**	**2005**	**12**	*2*	*3*	*1*	*1*	*2*	*1*	*2*
**Wu**	**2011a**	**15**	*2*	*3*	*2*	*2*	*2*	*1*	*3*
**Wu**	**2011b**	**9**	*1*	*3*	*2*	*1*	*0*	*1*	*1*
**Yang**	**2012**	**12**	*1*	*3*	*2*	*1*	*1*	*1*	*3*
**Yang**	**2008**	**11**	*1*	*3*	*2*	*2*	*1*	*1*	*1*
**Zhao**	**2018**	**10**	*1*	*3*	*2*	*1*	*1*	*1*	*1*
**Zheng**	**2016**	**11**	*1*	*3*	*2*	*2*	*0*	*1*	*2*
**Zhou**	**2008**	**9**	*1*	*3*	*2*	*1*	*0*	*1*	*1*
**Zhu**	**2018**	**12**	*1*	*3*	*2*	*2*	*1*	*1*	*2*
**Zhu**	**2008**	**10**	*1*	*3*	*2*	*2*	*0*	*1*	*1*
**Zhu**	**2007**	**11**	*1*	*1*	*2*	*2*	*1*	*1*	*3*

a, Selected from population cancer registry (2); Selected from hospital (1); No method of selection described (0).

b, Population-based (3); Blood donors (2); Hospital-based (1); Not described (0).

c, Histopathologic confirmation (2); by patient medical record (1); Not described (0).

d, Controls matched with cases by age and sex (2); Controls matched with cases only by age or by sex (1); Not matched or not descried (0).

e, Genotyping performed blindly and quality control (2); only genotyping performed blindly or quality control (1); Unblinded and without quality control (0).

f, HWE in the control group (1); HWD in the control group or not mentioned (0).

g, > 1000 (3); 501~1000 (2); 201~500 (1); ≤ 200 (0).

Table S4. Subgroup analysis data by the factors of country, genotyping assay and article quality.

Genetic model	Subgroup	Sample size		Association	
		Study	Case/control	$P_{association}$	OR (95% CI)
**T vs. C**	USA	20	9,117/11,690	0.642	1.01 (0.95,1.08)
	China	32	11,180/16,386	0.497	1.02 (0.96,1.09)
	PCR-RFLP	23	6,404/9,124	0.147	1.08 (0.97,1.19)
	TaqMan	19	7,886/11,733	0.948	1.00 (0.94,1.06)
	MassARRAY	15	5,224/7,253	0.165	1.08 (0.97,1.21)
	High quality	62	25,006/35,149	0.473	1.02 (0.97,1.08)
**TT vs. CC**	USA	20	9,117/11,690	0.201	1.09 (0.95,1.25)
	China	32	11,180/16,386	0.109	1.11 (0.98,1.25)
	PCR-RFLP	23	6,404/9,124	0.033	1.26 (1.02,1.55)
	TaqMan	19	7,886/11,733	0.287	1.07 (0.95,1.20)
	MassARRAY	15	5,224/7,253	0.286	1.16 (0.98,1.25)
	High quality	62	25,006/35,149	0.221	1.08 (0.89,1.51)
**CT vs. CC**	USA	20	9,117/11,690	0.394	0.96 (0.88,1.05)

	China	32	11,180/16,386	0.299	0.95 (0.88,1.04)
	PCR-RFLP	23	6,404/9,124	0.862	0.99 (0.88,1.11)
	TaqMan	19	7,886/11,733	0.019	0.92 (0.85,0.99)
	MassARRAY	15	5,224/7,253	0.340	1.06 (0.94,1.21)
	High quality	62	25,006/35,149	0.381	0.97 (0.91,1.03)
**CT+TT vs. CC**	USA	20	9,117/11,690	0.646	0.98 (0.90,1.06)
	China	32	11,180/16,386	0.738	0.98 (0.90,1.08)
	PCR-RFLP	23	6,404/9,124	0.557	1.04 (0.92,1.17)
	TaqMan	19	7,886/11,733	0.115	0.94 (0.88,1.01)
	MassARRAY	15	5,224/7,253	0.225	1.08 (0.95,1.24)
	High quality	62	25,006/35,149	0.853	0.99 (0.93,1.06)
**TT vs. CC+ CT**	USA	20	9,117/11,690	0.031	1.04 (1.01,1.27)
	China	32	11,180/16,386	0.006	1.04 (1.04,1.24)
	PCR-RFLP	23	6,404/9,124	0.005	1.28 (1.08,1.52)
	TaqMan	19	7,886/11,733	0.018	1.12 (1.02,1.23)
	MassARRAY	15	5,224/7,253	0.337	1.13 (0.88,1.45)
	High quality	62	25,006/35,149	0.067	1.09 (0.99,1.20)

OR, odds ratio; CI, 95% confidence interval; $P_{association}$, P value in the association test; PCR, polymerase chain reaction; RFLP, restriction fragment length polymorphism.

Table S5. Subgroup analysis data of different system cancers.

Genetic model	Subgroup	Sample size		Association	
		Study	Case/control	$P_{association}$	OR (95% CI)
**T vs. C**	urinary system cancer	8	3,460/3,613	0.004	1.25 (1.07,1.45)
	respiratory system cancer	10	2,642/6,319	0.222	1.05 (0.97,1.13)
	reproductive system cancer	6	1,907/2,480	0.166	0.89 (0.75,1.05)
	digestive system cancer	21	9,136/11,852	0.382	0.96 (0.87,1.06)
	head and neck cancer	5	1,327/1,875	0.078	1.20 (0.98,1.46)
**TT vs. CC**	urinary system cancer	8	3,460/3,613	0.001	1.68 (1.25,2.26)
	respiratory system cancer	10	2,642/6,319	0.252	1.11 (0.93,1.34)
	reproductive system cancer	6	1,907/2,480	0.442	0.87 (0.62,1.23)
	digestive system cancer	21	9,136/11,852	0.370	0.92 (0.75,1.11)
	head and neck cancer	5	1,327/1,875	0.119	1.48 (0.90,2.41)
**CT vs. CC**	urinary system cancer	8	3,460/3,613	0.069	1.17 (0.99,1.39)
	respiratory system cancer	10	2,642/6,319	0.368	1.05 (0.95,1.16)
	reproductive system cancer	6	1,907/2,480	0.006	0.81 (0.70,0.94)

	digestive system cancer	21	9,136/11,852	0.176	0.92 (0.83,1.04)
	head and neck cancer	5	1,327/1,875	0.883	1.02 (0.75,1.41)
**CT+TT vs. CC**	urinary system cancer	8	3,460/3,613	0.016	1.26 (1.04,1.53)
	respiratory system cancer	10	2,642/6,319	0.282	1.05 (0.96,1.16)
	reproductive system cancer	6	1,907/2,480	0.041	0.82 (0.68,0.99)
	digestive system cancer	21	9,136/11,852	0.255	0.93 (0.82,1.05)
	head and neck cancer	5	1,327/1,875	0.402	1.13 (0.85,1.49)
**TT vs. CC+ CT**	urinary system cancer	8	3,460/3,613	0.001	1.49 (1.18,1.90)
	respiratory system cancer	10	2,642/6,319	0.293	1.10 (0.92,1.32)
	reproductive system cancer	6	1,907/2,480	0.754	0.96 (0.72,1.26)
	digestive system cancer	21	9,136/11,852	0.601	0.96 (0.81,1.13)
	head and neck cancer	5	1,327/1,875	0.024	1.58 (1.06,2.34)

OR, odds ratio; CI, 95% confidence interval; $P_{association,}$ *P* value in the association test.

Table S6. False-positive report probability values for the association between *XPC* rs2228000 and the risk of bladder, gastric and breast cancer.

Cancer type	Model	OR (95% CI)	$P^{\$}$	Statistical power	Prior probability				
					0.25	0.1	0.01	0.001	0.0001
**bladder cancer**	**T vs. C**	1.25 (1.07,1.45)	0.003	0.992	**0.010**	**0.028**	0.243	0.764	0.970
	**TT vs. CC**	1.68 (1.25,2.26)	<0.001	0.227	**0.008**	**0.023**	0.209	0.728	0.964
	**CT+TT vs. CC**	1.26 (1.04,1.53)	0.020	0.961	**0.058**	**0.155**	0.669	0.953	0.995
	**TT vs. CC+ CT**	1.49 (1.18,1.90)	0.001	0.522	**0.007**	**0.022**	**0.198**	0.714	0.961
**breast cancer**	**T vs. C**	1.11 (1.02,1.21)	0.018	1.000	**0.050**	**0.138**	0.637	0.947	0.994
	**TT vs. CC**	1.33 (1.10,1.60)	0.013	0.912	**0.042**	**0.116**	0.590	0.936	0.993
	**TT vs. CC+ CT**	1.29 (1.12,1.48)	<0.001	0.984	**0.001**	**0.003**	**0.027**	0.222	0.740
**gastric cancer**	**T vs. C**	0.92 (0.85,0.99)	0.026	1.000	**0.072**	**0.189**	0.719	0.963	0.996
	**CT vs. CC**	0.83 (0.73,0.93)	0.001	1.000	**0.004**	**0.012**	**0.116**	0.570	0.930
	**CT+TT vs. CC**	0.84 (0.76,0.94)	0.002	1.000	**0.007**	**0.021**	**0.191**	0.704	0.960

OR, odds ratio; CI, 95% confidence interval; $P^{\$}$, *P* value in Chi-square test for genotype frequency distributions;

FPRP value < 0.2 in bold.
